# The CK1 Family: Contribution to Cellular Stress Response and Its Role in Carcinogenesis

**DOI:** 10.3389/fonc.2014.00096

**Published:** 2014-05-19

**Authors:** Uwe Knippschild, Marc Krüger, Julia Richter, Pengfei Xu, Balbina García-Reyes, Christian Peifer, Jakob Halekotte, Vasiliy Bakulev, Joachim Bischof

**Affiliations:** ^1^Department of General and Visceral Surgery, Surgery Center, Ulm University Hospital, Ulm, Germany; ^2^Institute for Pharmaceutical Chemistry, Christian Albrechts University, Kiel, Germany; ^3^Department of Organic Synthesis, Ural Federal University, Ekaterinburg, Russia

**Keywords:** casein kinase 1, cellular stress, centrosome, p53, signal transduction, tumorigenesis, inhibitor, disease

## Abstract

Members of the highly conserved and ubiquitously expressed pleiotropic CK1 family play major regulatory roles in many cellular processes including DNA-processing and repair, proliferation, cytoskeleton dynamics, vesicular trafficking, apoptosis, and cell differentiation. As a consequence of cellular stress conditions, interaction of CK1 with the mitotic spindle is manifold increased pointing to regulatory functions at the mitotic checkpoint. Furthermore, CK1 is able to alter the activity of key proteins in signal transduction and signal integration molecules. In line with this notion, CK1 is tightly connected to the regulation and degradation of β-catenin, p53, and MDM2. Considering the importance of CK1 for accurate cell division and regulation of tumor suppressor functions, it is not surprising that mutations and alterations in the expression and/or activity of CK1 isoforms are often detected in various tumor entities including cancer of the kidney, choriocarcinomas, breast carcinomas, oral cancer, adenocarcinomas of the pancreas, and ovarian cancer. Therefore, scientific effort has enormously increased (i) to understand the regulation of CK1 and its involvement in tumorigenesis- and tumor progression-related signal transduction pathways and (ii) to develop CK1-specific inhibitors for the use in personalized therapy concepts. In this review, we summarize the current knowledge regarding CK1 regulation, function, and interaction with cellular proteins playing central roles in cellular stress-responses and carcinogenesis.

## The CK1 Family

**Figure 1:**
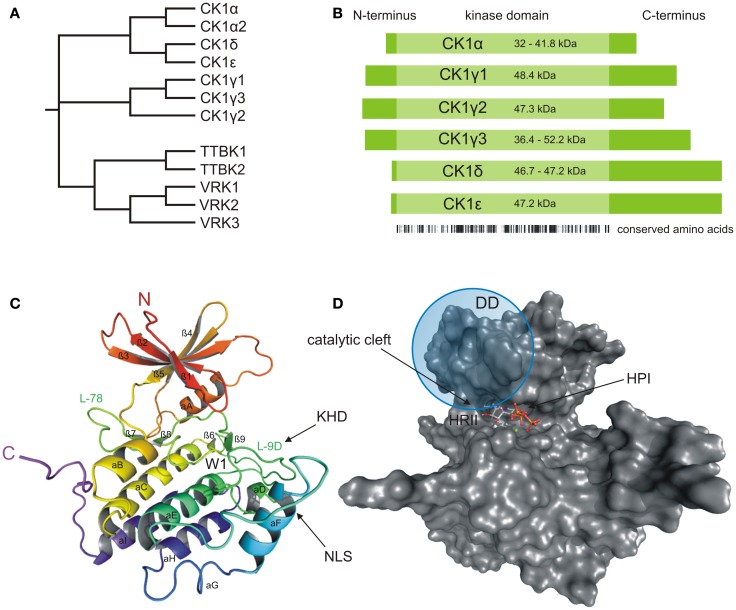
**Structural presentation of CK1δ**. **(A)** Phylogenetic relation between CK1 isoforms of *Homo sapiens* (CK1α, γ1–3, δ, and ε) and other members of the human CK1 family (TTBK1–2, VRK1–3). **(B)** Schematic alignment of human CK1 isoforms α, γ1–3, δ, and ε. Their molecular weight varies between 32 (CK1α) and 52.2 kDa (CK1γ3). In case transcription variants have been reported for one isoform, the molecular weight is given as range from the smallest to the largest variant. All CK1 isoforms are highly conserved within their kinase domains (light green box, 286 aa), but differ within their variable N- (4–40 aa) and C-terminal (39–122 aa) non-catalytic domains (dark green boxes) [according to Knippschild et al. (333)]. Ribbon **(C)** and surface **(D)** diagram of the molecular structure of CK1δ (PDB code 4HGT) modeled in complex with Mg^2+^-ATP at a resolution of 1.80 Å. The nomenclature is adapted from Xu et al. (24) and Longenecker et al. (25). Until today, crystal structures of human CK1 isoforms γ1 (PDB code 2CMW), γ2 (2C47), γ3 (2CHL, 2IZR, 2IZS, 2IZT, 2IZU, 4HGL, 4HGS, 4G16, 4G17), δ (4KB8, 4KBA, 4KBC, 4KBK, 4HNF, 3UYS, 3UYT, 3UZP), and ε (4HNI, 4HOK) are accessible as well. For reasons of clarity, we focused on CK1δ exemplarily, due to its superior relevance. The catalytic domain folds into two lobes primarily containing strands (N-terminal), respectively helices (C-terminal) forming a catalytic cleft between that represents the ATP binding pocket as well as a substrate binding site. KHD indicates the kinesin homology domain within L-9D. DD refers to a putative dimerization domain containing various amino acids of β1, β2, β5, L-5B, β7, and αB, whereas NLS displays a putative nuclear localization signal sequence at the junction between L-EF and αF. A tungstate molecule binding site identifies a specific phosphate moiety binding motif (W1). The active site contains a deep hydrophobic pocket (HPI) and a spacious hydrophobic region (HRII) (25–28). All modeling and docking studies were performed using Schrödinger software (Maestro, version 9.3, Schrödinger, LLC, New York, NY, 2012; Glide, version 5.8, Schrödinger, LLC, New York, NY, 2012). The illustration of modeling results was generated by the PyMOL Molecular Graphics System (Version 1.5.0.4, LLC) (29).

Members of the CK1 (formerly named casein kinase 1) family were among the first kinases described in literature (1). Although the milk protein component casein is not a physiological substrate for CK1, it reflects its preference for serine or threonine residues N-terminally flanked by already phosphorylated amino acid residues or acidic amino acids (2–7). Seven distinct genes encoding mammalian CK1 isoforms α, β, γ1, γ2, γ3, δ, and ε as well as various post-transcriptionally processed splice variants (transcription variants; TV) have been characterized (except for β all are expressed in humans). The closest relatives to the CK1 family are tau tubulin kinases 1 and 2 (TTBK1/2) and the vaccinia-related kinases 1–3 (VRK1-3) (Figure 1A). All CK1 isoforms are highly conserved within their kinase domains (51–98% identical) while the highly related isoforms CK1δ and ε display the highest homology. However, CK1 family members differ significantly in length and primary structure of their regulatory non-catalytic C-terminal domains, resulting in molecular weights ranging from 32 kDa (CK1α) to 52.2 kDa (CK1γ3) (Figure 1B) (5, 8–16). Meanwhile, CK1 homologous proteins have also been isolated from yeast, basidiomycetes, plants, algae, and protozoa (9, 15, 17–23). Since recognition motifs for CK1 are found on most cellular proteins, more than 140 *in vitro* and *in vivo* substrates have been reported thus far (see CK1 Substrate Specificity and Table 1). Therefore, in a cellular context a tight regulation of CK1 activity and expression is indispensable. Known general mechanisms for CK1 regulation include (i) phosphorylation by inhibitory autophosphorylation and/or (ii) phosphorylation by other cellular protein kinases, and (iii) interaction with cellular proteins or subcellular sequestration (see Regulation of CK1 Activity). Based on the broad spectrum of target proteins, CK1 family members are involved in modulating a variety of cellular functions: in immune response and inflammation (see CK1 in Immune Response and Inflammation), in spindle and centrosome-associated processes (see Interaction of CK1 with Centrosomes, Tubulin, and Microtubule-Associated Proteins), in DNA damage-related signal transduction (see CK1 in DNA Damage-Related Signal Transduction), in circadian rhythm (see CK1 in Circadian Rhythm and its Connections to Stress Response), and in apoptosis (see CK1-Signaling in Apoptotic Pathways). Consequently, a deregulation or dysfunction of CK1 in pathways responsible for regulation of growth, proliferation, and apoptosis may result in pathological conditions (see CK1 and the Wnt Pathway, CK1 in the Hedgehog Pathway to CK1 in the Hippo Pathway), such as tumorigenesis (see CK1-Related Tumorigenic Functions and CK1 in Metastatic Processes) or neurological diseases. Therefore, interest in CK1 isoforms as new drug targets has enormously increased within the last 15 years and led to development of several CK1-specific inhibitors (see CK1-Specific Inhibitors).

**Table 1:** **Reported substrates for CK1 family members and reported *in vitro* and *in vivo* substrates of CK1 family members of several species**.

Functional groups	CK1 substrates
Cytoskeleton-associated proteins, adhesion factors, and scaffolding proteins	Myosin (56), troponin (56), ankyrin (57), spektrin 3 (58), filamin (59), vinculin (59), neurofilamentary proteins (60, 61), dynein (62), α-/β-tubulin (32), microtubule-associated protein (MAP) 1A (63), MAP 4 (32), stathmin (32), tau (32, 64), keratin 17 (65), desmolein (65), annexin II (65), centaurin-α (p42IP4) (66, 67), neural cell-adhesion molecule (NCAM) (68), E-cadherin (69), RhoB (70), myelin basic protein (MBP) (55), kinesin-like protein 10A (KLP10A) (71), lectin L-29 (72), galectin-3 (73), end binding 1 (EB1) (74), Sid4 (75), connexin-43 (76), metastasis suppressor 1 (MTSS1) (77), and Hsp79 and Hsp90 (78)
Receptors	β-Subunit of the insulin-receptor (79), TNFα-receptor (80), muscarin M3-receptor (81), Ste2p (α-factor-receptor) (82), Ste3p (α-factor-receptor) (83), platelet derived growth factor (PDGF) receptor (84), retinoid X receptor (RXR) (85), low density lipoprotein-related receptor protein (LRP) 6 (86, 87), type I interferon receptor (IFNAR1) (88), estrogen receptor α (ERα), amplified in breast cancer 1 (AIB1) (89), calmodulin (CaM) (90), and Ror2 (91)
Membrane transporters	Erythrocytes anion transporter (92), uracil permease (*Saccharomyces cerevisiae*) (93), translocase of the outer mitochondrial membrane 22 (Tom22) (94), and α-T663-hENaC (95)
DNA-/RNA-associated proteins	Non-histone chromatin proteins (96), RNA polymerase I and II (97), topoisomerase IIα (98), Star-poly(A) polymerase (Star-PAP) (99), Rec8 (100), DNA methyl-transferase (Dnmt1) (101), TAR DNA-binding protein of 43 kDa (TDP-43) (102), DEAD-box RNA helicase DDX3 (103), Ubiquitin-like, with PHD, and RING finger domains 1 (UHRF1) (104)
Ribosome-related proteins	15 kDa (105), 20 kDa (105), 35 kDa (105), L4 (65), L8 (65), L13 (65), ribosomal protein S6 (rpS6) (106), and ENP1/BYSL and LTV1 (107)
Transcription and splice factors	p53 (108), cyclic AMP responsive element modulator (CREM) (109), Swi6 (110), nuclear factor of activated T-cells (NFAT) (111), serine/arginine-rich (SR) proteins (112), T-cell factor (Tcf) 3 (113), brain and muscle Arnt-like protein (BMAL) 1 (114), cryptochrome 1 (CRY) (114), β-catenin (115, 116), armadillo (117), SMAD 1–3 and 5 (118), osmotic response element-binding protein (OREBP) (119), cubitus interruptus (Ci) (120), forkhead box G1 (FoxG1) (121), SNAIL (122), tafazzin (TAZ) (123), yes-associated protein (YAP) (124), proliferator-activated receptor γ co-activator 1α (PGC-1α) (125), *Drosophila* Myc (d-Myc) (126), cyclic AMP response element-binding protein (CREB) (127), Sre1N (yeast sterol regulatory element-binding protein homolog) (128), and NFκB (nuclear factor “kappa-light-chain-enhancer” of activated B-cells) subunit p65 (129)
Translation factors	Initiation factors (IF) 4B (130), 4E(5, 6, 130, 131)
Viral proteins	Simian virus 40 large T-antigen (SV40 T-Ag) (132), hepatitis C virus non-structural 5A (NS5A) (133), human cytomegalovirus ppUL44 (134), Poa semilatent hordeivirus triple gene block 1 (TGB1) (135), Kaposi sarcoma-associated herpesvirus latency associated nuclear antigen (LANA) (136), and yellow fever virus methyl-transferase (137)
Kinases and phosphatases	Cyclin-dependent kinase 5 (Cdk5) (138), protein kinase C (PKC) (139), protein kinase D2 (PKD2) (140), cell division cycle 25 (Cdc25) (141–143), and PH domain and leucine rich repeat protein phosphatase 1 (PHLPP1) (144)
Inhibitors and modulators	Inhibitor 2 of PPA 1 (145, 146), dopamine and cAMP regulated phosphoprotein of 32 kDa (DARPP-32) (147), disheveled (148), mammalian period circadian protein (mPER) (149), adenomatous polyposis coli (APC) (150), Bid (151), protein kinase C potentiated myosin phosphatase inhibitor of 17 kDa (CPI-17) (152), nm23-H1 (153), 14-3-3 proteins (154), MDM2 (155), MDMX (156), FREQUENCY (FRQ) (157), WHITE COLLAR-1 (WC-1) (158), CARD containing MAGUK protein (CARMA1)/caspase recruitment domain (CARD11) (159), SLR1 (160), endogenous meiotic inhibitor 2 (Emi2) (161), Chk1-activating domain (CKAD) of claspin (162), PER2 (163), protein S (164), Rap guanine nucleotide exchange factor 2 (RAPGEF2) (165), and Sprouty2 (SPRY2) (166)
Enzymes (miscellaneous)	Acetyl-CoA carboxylase (167), glycogen synthase (168, 169), yeast endoprotease Ssy5 (170), and neural precursor cell expressed developmentally down-regulated protein 4 (Nedd4) (171)
Vesicle- and trafficking-associated proteins	SV2 (172), β3A- and β3B-subunit of the AP-3 complex (173), snapin (174), and ceramide transfer protein (CERT) (175)
Receptor-associated proteins	Fas-associated death domain (FADD) (176), receptor interacting protein 1 (RIP1) (177)
Factors of neuro-degenerative diseases	Presenilin-2 (178), tau (64), β-secretase (179), parkin (180), and α-synuclein (181)
Metastatic tumor antigens	Metastatic tumor antigen 1, short form (MTA1s) (182)

### CK1 structure and domains

**Figure 2:**
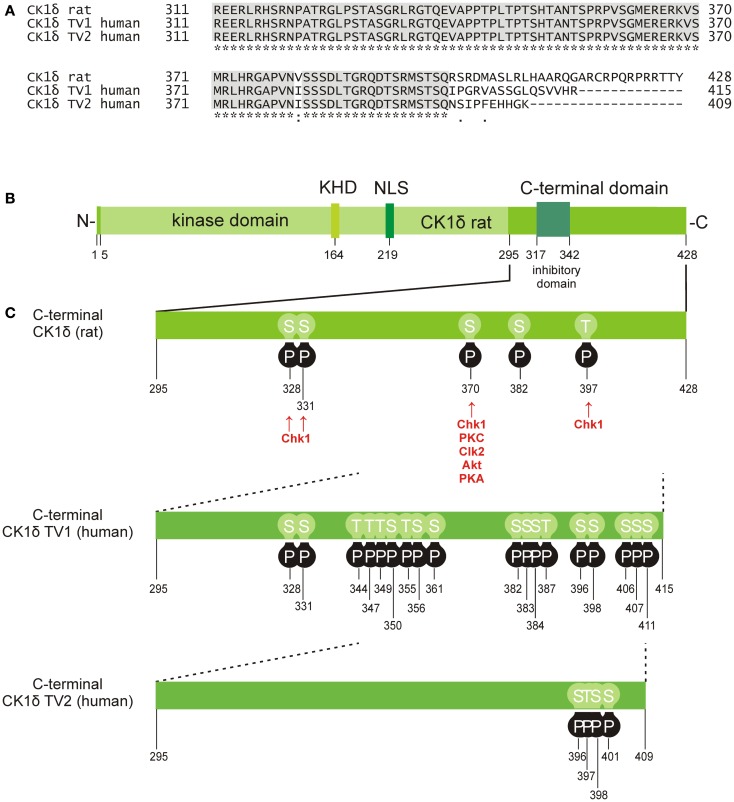
**Phosphorylation sites located in the C-terminal domain of CK1δ**. **(A)** Alignment of the rat CK1δ C-terminal sequence with the human CK1δ transcription variants 1 and 2 C-terminal sequences (accession numbers L07578, NM001893, and NM139062, respectively) generated by using the program ClustalW (36, 37), showing conserved amino acids (gray) and obvious differences in the C-terminal domain beyond amino acid 399. **(B)** Domain structure of rat CK1δ (NLS: nuclear localization signal, KHD: kinesin homology domain). **(C)** Phosphorylation sites in the C-terminal regulatory domains of CK1δ rat and human transcription variants 1 and 2, that have so far been confirmed experimentally (38–53). Kinases identified for phosphorylation of the C-terminal domain are shown for rat CK1δ (38, 39).

As a member of the superfamily of serine/threonine-specific kinases, CK1 represents the typical bi-lobal structure, which includes a smaller N-terminal lobe, primarily consisting of β-sheets, and a larger, mainly α-helical C-terminal lobe. The two lobes are connected by a hinge region forming a catalytic cleft for substrate and ATP binding (Figures 1C,D) (24, 25). In comparison to the general structural features of protein kinases, a prominent α-helix (αA-helix) within the N-terminal region is crucial for conformational regulation of kinase activity. A conserved glycine-rich loop (P-loop, bridging strands β1 and β2) forms the ceiling of the ATP active site and contributes to coordination of the γ-phosphate moiety of ATP (30). Contributing to structure-based inhibitor design, another loop (L-78) in close proximity to the hinge region has been demonstrated to trigger CK1 inhibitor selectivity (31). Within the C-terminal region, a specific phosphate moiety binding motif (W1) has been identified affording recognition of phosphorylated protein substrates and is further believed to be involved in CK1 regulatory interactions (9, 24, 25). In addition, a kinesin homology domain (KHD) within the T-loop (L-9D) and a putative dimerization domain (DD, containing various amino acids of strands β1, β2, β5, hinge region, β7, and αB) can be found inside the catalytic domain of CK1δ (Figures 1C and 2) (26, 32–34). The KHD is thought to support the interaction of CK1 isoforms with components of the cytoskeleton as this domain has been shown to be necessary for the interaction of kinesins with microtubules (MT) (26, 32–34). Furthermore, a putative nuclear localization signal sequence (NLS) at the junction between L-EF and αF has been reported to affect substrate binding (Figure 1C). The present NLS however seems to be not sufficient for nuclear localization of CK1δ because only CK1αL variants, carrying an additional NLS in the L-exon, are able to localize to the nucleus (35).The L-9D loop represents the homolog of the so-called activation-loop identified in other protein kinases and may therefore play a role in CK1 regulation. Moreover, loops L-9D and L-EF may be of importance in substrate recognition (Figure 1C) (24–27). The ATP active site itself mainly consists of a deep hydrophobic pocket (HPI, selectivity pocket) lined by the gatekeeper (Met-82 in CK1δ) and a second spacious hydrophobic region (HRII) adjacent to the hinge region as well as sugar and phosphate binding domains (Figure 1D) (31).

### CK1 substrate specificity

Belonging to the group of acidotropic protein kinases, CK1 family members mainly recognize substrates containing acidic or phosphorylated amino acid residues. The canonical consensus sequence for CK1 protein kinases is represented by the motif pSer/Thr-X-X-(X)-Ser/Thr whereas pSer/Thr indicates a phosphorylated serine or threonine residue. However, CK1 not only relies on phospho-primed motifs since the phospho-serine or phospho-threonine can also be replaced by an agglomeration of negatively charged acidic amino acids (2–7). In addition, non-canonical consensus sequences for CK1 family members have been described such as the SLS motif, found in β-catenin and nuclear factor of activated T-cells (NFAT), or the motif Lys/Arg-X-Lys/Arg-X-X-Ser/Thr occurring in sulfatide and cholesterol-3-sulfate (SCS) binding proteins (54, 55). Generally, substrate recognition motifs for CK1 protein kinases are massively distributed on cellular proteins. At present, more than 140 *in vitro* and *in vivo* substrates for CK1 isoforms have been reported, underlining its pleiotropic character (Table 1).

### Regulation of CK1 activity

Although members of the CK1 family are ubiquitously expressed, their expression levels differ depending on tissue and cell type (34, 183, 184). Certain factors seem to change the expression and activity of CK1, such as stimulation with insulin (185) or gastrin (140), viral transformation (186), treatment with topoisomerase inhibitors or other small molecules like calotropin (187), γ-irradiation (188), or altered membrane concentrations of phosphatidylinositol-4,5-bisphosphate (PIP2) (172). At the protein level, certain mechanisms regulating CK1 activity have been identified: structure-related regulation, subcellular localization, interaction with other proteins, and post-translational modifications.

In X-ray crystallography, CK1δ was found to form dimers. In the dimeric form, the adenine binding domain is occupied by the specific intramolecular contacts of the dimerization domain. As a consequence, ATP is excluded from the active center of the kinase. Therefore, formation of homodimers could possibly have a negative regulatory effect on CK1δ kinase activity *in vivo* (26). This hypothesis is supported by further observations: the expression of a mutant CK1δ with impaired kinase activity lead to down-regulation of endogenous CK1δ activity in a dominant-negative way in simian virus 40 (SV40)-transformed cell lines as well as to changes in mammary tumorigenesis in WAP-mutCK1δ/WAP-T bi-transgenic mice (189, 190).

Appropriate sequestration of CK1 proteins to particular cellular compartments is crucial for access to their pool of substrates (21, 191, 192). As an example, in *Saccharomyces cerevisiae* kinase activity of C-terminal deletion mutants of membrane-bound YCK1 and YCK2 could only be rescued by replacing the nuclear localization signal of the CK1 homolog Hrr25 with a prenylation motif, which is required for plasma membrane localization. Conversely, loss of Hrr25 function after deletion of its NLS could only be rescued by replacing the prenylation motif in YCK1 and YCK2 with a NLS. These observations led to the conclusion that merely partial cellular overlap of these three isoforms is not enough to rescue the deletion phenotype (192). In experiments using a CK1δ kinase-dead mutant, it has been shown that not only the existence of the kinase domain, but also the catalytic activity of the protein is essential for its appropriate subcellular localization (193). Additionally, a study designed to identify binding partners, which recruit CK1 to Alzheimer’s disease (AD) ubiquitinated lesions identified a dysbindin structural homolog that interacts with CK1γ, δ, and ε, and in the case of CK1δ it has been shown to be a concentration-dependent inhibitor (194).

It is very common to find certain motifs in proteins that act as scaffolds, which direct the proper positioning of protein complexes. It has also been suggested that such scaffolds additionally exert complex allosteric control of their partners thereby regulating their activity [reviewed in Cheong and Virshup (195) and Good et al. (196)]. In general, proteins that function as scaffolds tether members of signaling pathways into complexes thereby increasing the interaction efficiency between partner molecules (196, 197). In the case of CK1, these scaffolds have an important regulatory role because they might change the affinity of CK1 isoforms for their substrates as well as the rate of phosphorylation and activation of CK1 kinase activity over the basal level (196, 198). In fact, protein scaffolds have been already found to exert substantial control over different kinase-mediated signaling pathways [reviewed in Brown et al. (199)], though they are not limited to the coordination of kinase cascades (196). Examples for such protein scaffolds include the centrosomal and Golgi N-kinase anchoring protein (CG-NAP), also known as A-kinase anchoring protein 450 (AKAP450) (191) and the DEAD-box RNA helicase DDX3, which has been previously identified as scaffolding adaptor that directly activates the kinase IκB (200). AKAP450 specifically interacts with CK1δ and ε and recruits them to the centrosome, where they can exert centrosome-specific functions coupled to the cell cycle. This interaction is confirmed by the ability of AKAP450 to re-localize CK1δ at the plasma membrane, when it itself is attached to the membrane (191). Recently, it has been suggested that the interaction of CK1δ with AKAP450 is necessary to mediate primary ciliogenesis (201). In addition, evidence is increasing that in Wnt-signaling CK1 activity depends on DDX3 as a co-factor. DDX3 directly interacts with CK1ε in a Wnt-dependent manner, and promotes phosphorylation of Disheveled (DVL) (103). DDX3 can therefore be seen as regulatory subunit of CK1 isoforms with the potential to increase the activity of CK1α, γ2, δ, and ε by up to five orders of magnitude (103). Since CK1 isoforms have been shown to phosphorylate DDX3, it could be speculated that CK1 isoforms might also play a role in regulating the functions of DDX3 (103).

Finally, CK1 activity can furthermore be regulated by post-translational modifications, mainly represented by reversible phosphorylation either through autophosphorylation or site-specific phosphorylation mediated by cellular kinases. Within the regulatory C-terminal domains of CK1δ and ε, sequences with the motif pSer/Thr-X-X-Y (Y: any amino acid except serine or threonine) can be generated by autophosphorylation events and can consecutively act as pseudo-substrates blocking the catalytic center of the kinase (202–205). By using CK1δ truncation mutants, Ser-318, Thr-323, Ser-328, Thr-329, Ser-331, and Thr-337 were detected as candidate sites for intramolecular autophosphorylation. Although not all of them influenced kinase activity, truncation of the C-terminal part up to amino acid (aa) 317 significantly enhanced activity of CK1δ (204). For CK1ε amino acid residues Ser-323, Thr-325, Thr-334, Thr-337, Ser-368, Ser-405, Thr-407, and Ser-408 within the C-terminal domain are considered to be potential autophosphorylation sites (203). C-terminal inhibitory autophosphorylation could also be demonstrated for CK1γ1-3 as well as for CK1α and its splice variants CK1αL and CK1αS (16, 206).

Apart from intramolecular autophosphorylation, CK1 isoforms are also phosphorylated by other kinases. In the case of CK1δ, phosphorylation by PKA (cAMP-dependent protein kinase), Akt (protein kinase B), CLK2 (CDC-like kinase 2), protein kinase C isoform α (PKCα), and Chk1 (checkpoint kinase 1) has been demonstrated (38, 39) (Figure 2). PKA could be further characterized as a major CK1δ C-terminal targeting kinase predominantly phosphorylating Ser-370 both *in vitro* and *in vivo*. Mutation of Ser-370 to alanine increased kinase activity *in vitro* and enhanced formation of an ectopic dorsal axis during embryonic development of *Xenopus laevis* (39). More recently, Chk1 has been demonstrated to phosphorylate CK1δ at serine residues 328, 331, and 370, as well as threonine residue 397. Mutations at these sites proved to significantly increase kinase activity (38). Moreover, several residues in the C-terminal domain of CK1δ were found in a phosphorylated state in large-scale mass spectrometry analyses. However, the kinases responsible for the detected phosphorylation events were not specified (Figure 2 and references therein).

Generally, dephosphorylation of CK1 by serine/threonine-specific protein phosphatases or low levels of H_2_O_2_ result in an increase of kinase activity (202, 203, 207). Proteolytic cleavage of the C-terminal domain also results in multiple increase of CK1 kinase activity *in vitro* (28, 202, 204). In addition, neddylation of CK1α seems to be involved in CK1 regulation (208).

## CK1 in Stress-Related Cellular Functions

In response to stress situations like mechanical damage, toxin exposure, or environmental stress exposure, cells experience a variety of molecular changes, which are generally referred to as cellular stress response. The purpose of these changes is to protect the cell against conditions, which may cause acute damage, but also to build some kind of resistance toward long term unfavorable conditions. In response to extreme temperature or toxic substances, expression of heat shock proteins (Hsp) is transcriptionally increased. Most of these proteins belong to a group of proteins, which are involved in the (un-)folding of other proteins (209). A quite recent report links phosphorylation events mediated by CK1, CK2, and GSK3β to the regulation of Hsp70 and Hsp90. In more detail, phosphorylation of Hsp70 and Hsp90, mediated by these kinases, plays an important role in regulating their binding to co-chaperones like HOP (protein folding activity) and CHIP (ubiquitin ligase activity). In highly proliferative cells, phosphorylated Hsp70 and 90 form complexes with HOP whereas CHIP-binding is prevented by phosphorylation of Hsp70 and 90. Therefore, CK1, CK2, and GSK3β together with the action of phosphatases might be involved in complex regulation of the C-terminal phosphorylation of Hsp70 and Hsp90 and their binding to co-chaperones (78). Moreover, apart from environmental or external stress conditions, cells may also be challenged by stress originating from pathological conditions as in the case of inflammatory or proliferative diseases. A detailed presentation of CK1 isoforms in regulating cellular stress response can be found in the following chapters.

### CK1 in immune response and inflammation

By analyzing lymphatic tissues of BALB/c mice, remarkable immunoreactivity of CK1δ and ε in granulocytic and megakaryotic cells as well as in a subpopulation of lymphocytes has been detected (183, 184, 210). Mitogenic activation of T-lymphocytes was accompanied by a significant increase in both CK1δ protein levels and kinase activity (210).

So far, several mechanisms have been reported by which CK1 isoforms might be involved in regulating lymphocyte activation and granulocyte physiology. Transcriptional activators of the NFAT family of proteins play a major role in T-cell activation. Their translocation to the nucleus can be blocked by phosphorylation of numerous sites present in the NFAT regulatory domain (211). Some of these are phosphorylated by various CK1 isoforms (rat liver CK1 and *Danio rerio* CK1α) with high efficiency. In a two-phase phosphorylation mechanism, first phosphorylation of the non-canonical site Ser-177 is initiated by CK1 binding to a cluster of acidic residues within the sequence of aa 173–218. This event enhances the subsequent phosphorylation of downstream residues in a hierarchical manner (212). In contrast, Okamura and colleagues reported NFAT1 to be phosphorylated by CK1 within the serine-rich region SRR-1 (aa 149–183) after binding of CK1 to a N-terminal motif between aa 1–98 (213).

Upon T-cell receptor engagement dynamic association of CK1α to the CBM (CARMA/BCL10/MALT1) complex has been shown. This complex acts as an NFκB (nuclear factor “kappa-light-chain-enhancer” of activated B cells) activating platform containing the scaffold protein CARMA1, the adaptor protein BCL10, and the paracaspase MALT1. Here, CK1α complex association is linked to NFκB activation, increased cytokine production, and lymphocyte proliferation. However, CK1α was found to be a bi-functional regulator of NFκB signaling since phosphorylation and subsequent inactivation of CARMA1 leads to termination of receptor-induced NFκB activation (159). Just recently, CK1γ1 has been demonstrated to be a negative regulator in innate immunity by directly phosphorylating the NFκB subunit p65 following RIG-I pathway stimulation after RNA virus infection. This phosphorylation event is sufficient to target p65 for its degradation (129). Following immune receptor engagement a signal transduction platform is assembled around the T-cell receptor. This specialized cell–cell junction is known as the immunological synapse whose formation also leads to remodeling of the actin cytoskeleton and to repositioning of the centrosome to the immunological synapse (214). Herein, the polarization process is supported by CK1δ phosphorylating the microtubule plus-end-binding protein 1 (EB1). Formation of CK1δ–EB1 complexes is associated with increased speed of microtubule growth and most likely also with subsequent centrosome translocation in activated T-cells (74).

In granulocytes as well as in solid tumors cell survival is significantly promoted by the transcriptional activator HIF-1 (hypoxia-inducible factor-1), which is able to respond to changes in cellular oxygen levels. HIF-1 is continuously produced and marked for degradation by a hydroxylation step involving oxygen-dependent hydroxylases. Under hypoxic conditions, the continuous destruction of HIF-1 is blocked (215). Additionally, HIF-1 expression and activity can be regulated by oxygen-independent mechanisms resulting in phosphorylation of critical residues in HIF-1 regulatory domains. CK1δ has been identified as one of these kinases able to phosphorylate Ser-247 in the PAS-B (Per-ARNT-Sim-B) domain of HIF-1α. This modification has no effect on HIF-1α stability but affects the formation of the transcriptionally active HIF-1α-ARNT heterodimer, which is seen as an obligatory step prior to DNA binding (216). Therefore, active CK1δ can be seen as negative regulator of HIF-1-mediated cell survival.

Additionally, for the highly CK1δ homologous isoform CK1ε, a major role for transcriptional regulation in granulocytes has been suggested. Along with human granulocytic differentiation, a down-regulation of CK1ε has been observed. Here, active CK1ε was shown to interact with and to stabilize SOCS3 (suppressor of cytokine signaling 3) leading to attenuation of STAT3. Consequently, overexpression of CK1ε inhibited granulocyte-colony-stimulating factor (G-CSF) induced differentiation of myeloid progenitor cells (217).

### Interaction of CK1 with centrosomes, tubulin, and microtubule-associated proteins

Members of the CK1 family represent central components in the regulation of several cellular functions linked to cell cycle progression, spindle-dynamics, and chromosome segregation. CK1α has been shown to be located at the centrosome, microtubule asters, and the kinetochore (218–220). In addition, CK1δ especially associates with the spindle apparatus during mitosis and directly modulates MT by phosphorylation of α-, β-, and γ-tubulin, thereby exerting stress-induced functions at the spindle apparatus and the centrosome (221, 222). Recently, knockdown of CK1δ by siRNA was reported to inhibit microtubule nucleation at the Golgi apparatus (201). Furthermore, homologs of CK1, such as casein kinase 1-like 6 (CKL6), associate with cortical MT *in vivo* and phosphorylate tubulin *in vitro* (223).

In addition to the direct interaction of CK1 with MT, their polymerization and stability can also be regulated by CK1-mediated phosphorylation of microtubule-associated proteins (MAPs) (224). CK1δ regulates microtubule- and spindle-dynamics in response to genotoxic stress in order to maintain genomic stability by site-specific phosphorylation of tubulin, stathmin, and the MAPs MAP4, MAP1A, and tau (32, 63, 219, 225–227) as well as the phosphorylation of Sid4 that delays cytokinesis (75). An abnormal hyperphosphorylation of tau by CK1δ can lead to microtubule destabilization and is associated with the pathogenesis of AD (220, 225, 227).

**Figure 3:**
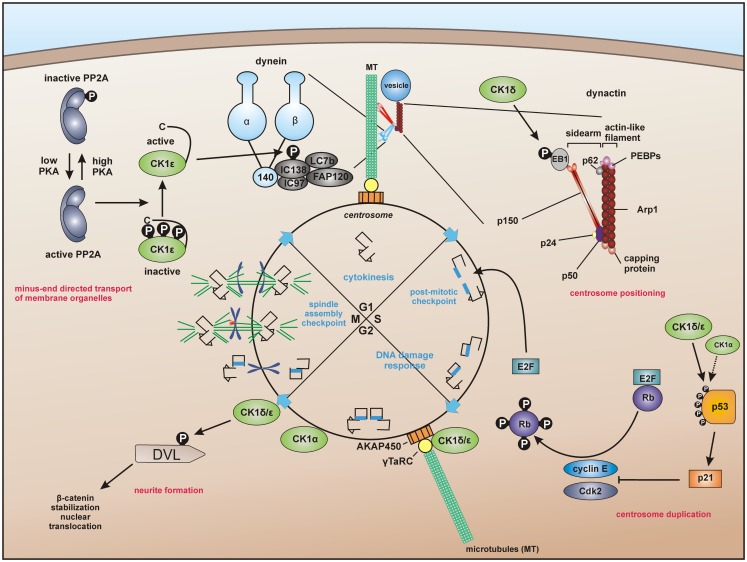
**Centrosome-associated functions of CK1**. For dynein-dependent transport along microtubules (MT), CK1ε phosphorylates the dynein intermediate chain (DIC) of dynein, likely IC138, thereby activating minus-end directed transport of membrane organelles along MT (62, 228). CK1δ and CK1ε are associated with the centrosome mediated through interaction with the scaffold protein AKAP450 (A-kinase anchor protein 450) (191, 193, 203). Both isoforms are related to Wnt-signaling and neurite outgrowth by phosphorylation of DVL (229, 230). In addition, CK1δ phosphorylates the end binding protein 1 (EB1), which is relevant for centrosome positioning during T-cell activation (74). Furthermore, a subpopulation of p53 in coordinated function with CK1 at the centrosome could ensure the integrity of the centrosome and thereby maintain genomic stability (231–233).

Recent studies provide evidence that CK1 influences dynein-dependent transport along MT. For instance, CK1ε phosphorylates dynein intermediate chain (DIC) of the motor protein dynein thereby activating minus-end directed transport of membrane organelles along MT and regulating dynein activity by phosphorylation of the DIC component IC138 (Figure 3) (62, 228).

A particular interesting role of centrosome-associated CK1 has been proposed in regulating cell cycle progression by interaction with the Wnt pathway and p53 (Figure 3). CK1δ is associated to the centrosome and related to Wnt3-dependent neurite outgrowth. In this context, phosphorylation of DVL by centrosome-associated CK1δ facilitates neurite formation (32, 193, 229). CK1δ co-localizes with DVL2 at basal bodies and gradually accumulates at centrosomes when cells proceed through the cell cycle (230). The hypothesis of CK1 fulfilling regulatory roles at the centrosome is further underlined by the already discussed findings that CK1δ and ε are anchored at the centrosome through interaction with AKAP450 (see Regulation of CK1 Activity) (191) and that CK1δ phosphorylates EB1, which is relevant for centrosome positioning during T-cell activation (see CK1 in Immune Response and Inflammation) (74). Remarkably, further studies revealed that a subpopulation of p53 is located at the centrosome in order to prevent genomic instability. Therefore, the coordinated function of both CK1 and p53 could ensure the integrity of the centrosome and thereby maintain genomic stability (231–233).

### CK1 in DNA damage-related signal transduction

CK1 family members can be considered as central components within the regulation of several cellular functions linked to DNA-processing or DNA damage [reviewed in Knippschild et al. (219)]. In context of DNA damage-associated signal transduction, p53 is activated initiating the activation of pathways ensuring centrosome integrity and genomic stability. This signaling network essentially involves coordinated action of CK1 and p53 (187, 231–233).

**Figure 4:**
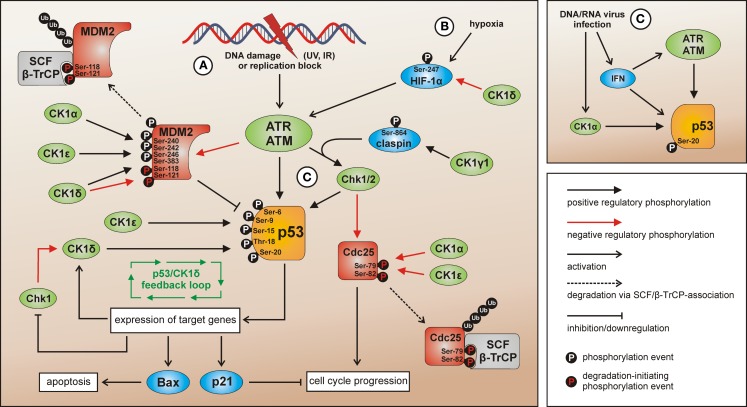
**CK1 isoforms in DNA damage-induced signal transduction**. After induction of DNA damage (situation A) p53 and Chk1/2 are activated by ATR/ATM-mediated phosphorylation while the p53-regulatory component MDM2 is inhibited. The activation of Chk1 is supported by claspin whereas Chk1/claspin-binding is promoted by CK1γ1-mediated phosphorylation of claspin (162). The CK1 isoforms α, δ, and ε are able to activate p53 by site-specific phosphorylation (187, 234, 235, 237). Activated p53 in turn induces the expression of target genes like Bax (leading to apoptosis), p21 (leading to cell cycle arrest), and also CK1δ (autoregulatory feedback loop) (187). MDM2-mediated degradation of p53 can be activated via interaction with and phosphorylation by CK1α, but also through phosphorylation by CK1δ or ε leading to enhanced binding of MDM2 to p53. CK1δ-mediated phosphorylation of Ser-118 and Ser-121 however marks MDM2 for proteasomal degradation (155, 239–241). In case Chk1/2 gets activated after DNA damage the phosphatase Cdc25, normally initiating cell cycle progression, is blocked by inhibitory phosphorylation and subsequent degradation. In the regulation of Cdc25 inhibition and degradation also CK1 isoforms α and ε are involved (141, 143). Signaling mediated by p53 can also be initiated by hypoxia (via CK1δ-regulated HIF-1α; situation B) (216, 243, 244) or DNA/RNA virus infection (via IFN and/or CK1α-related signal transduction; situation C) (236, 245). Depicted phosphorylation events refer to reported CK1-specific target sites.

CK1α, δ, and ε are able to phosphorylate certain N-terminal target sites of p53 (Ser-6, Ser-9, Ser-15, Thr-18, and Ser-20) (187, 234–237). By phosphorylation of p53 (mostly at Ser-15 and Thr-18) CK1δ and ε decrease p53 binding affinity to its cellular counterpart Mouse double-minute 2 homolog (MDM2) resulting in increased levels of MDM2-released, active p53 (234, 238, 239). Conversely, phosphorylation of MDM2 at several serine residues within its central acidic domain (Ser-240, Ser-242, Ser-246, and Ser-383) results in increased MDM2-p53-binding and subsequent degradation of p53 under non-stress conditions. Phosphorylation of Ser-118 and Ser-121 by CK1δ, however, can mark MDM2 for SCF/β-TrCP (Skp1, Cullins, F-box/β-transducin repeat containing E3 ubiquitin protein ligase) binding and ubiquitination, finally leading to proteasomal degradation of MDM2 (Figure 4) (155, 239–241). Under normal conditions, CK1α has furthermore been suggested to be a key player promoting p53 inhibition and degradation by MDM2. Therefore, CK1α is physically interacting with MDM2 resulting in p53 degradation. Inhibition or depletion of CK1α as well as inhibition of CK1α-MDM2 association leads to p53 stabilization (208, 242). For the MDM2 homolog MDMX, phosphorylation of Ser-289 by CK1α has been confirmed resulting in increased binding to p53 and subsequent inhibition of p53 transcriptional function (156).

Among the target genes activated by p53 following genotoxic stress also transcription of CK1δ can be induced (187). Given the previously discussed fact that p53 can be activated by CK1δ-mediated phosphorylation in this network, an autoregulatory feedback loop between CK1δ and p53 has been suggested (Figure 4).

Apart from DNA damage, p53 activation can also be induced by hypoxia. Herein, p53 levels are stabilized via HIF-1α and its positive regulatory effect on ATM/ATR (ataxia telangiectasia-mutated/ataxia telangiectasia and Rad3-related) (243, 244). As discussed previously, HIF-1α represents a substrate for CK1 and its transcriptional activity can be negatively regulated by CK1δ-mediated phosphorylation (216). However, since this modification has no effect on HIF-1α protein stability, the precise role of CK1δ-mediated HIF-1α phosphorylation in regulating ATM/ATR- and p53-specific functions under hypoxic conditions remains to be characterized.

More recent work suggested that interferon (IFN)-related signaling is able to activate p53 as a response to loss of epigenetic gene silencing (246). Among other critically involved epigenetic regulators, UHRF1 (ubiquitin-like, with PHD and RING finger domains 1) regulates the maintenance of DNA methylation during DNA replication (247). The stability of UHRF1 is regulated by proteasomal degradation including a priming step by CK1δ-mediated phosphorylation of Ser-108 thereby creating a recognition site for the SCF/β-TrCP ubiquitin ligase (104). Consequences of this negative regulatory connection between CK1δ and UHRF1 may also include the loss of stable DNA methylation and IFN-dependent activation of p53.

DNA/RNA virus infection has been described as a further mechanism resulting in p53 activation. This effect might be mediated via IFN-related p53 accumulation (245) but also via CK1-dependent signaling. In this context, CK1α-mediated phosphorylation of p53 at Ser-20 is induced after infection of T-cells with human Herpes virus 6B (HHV-6B). This phosphorylation event stabilizes the binding of p53 to the transcriptional co-activator p300. Therefore, CK1α takes part in gene regulation following virus infection induced p53 activation (236). Also infection with SV40 interferes with the p53 signaling network. SV40 large T-antigen (T-Ag) inactivates p53-dependent transcriptional activation whereas the oncogenic properties of T-Ag are enhanced by CK1-mediated phosphorylation (189, 190, 248). Moreover, as a consequence of SV40 infection/transformation, MDM2 is metabolically stabilized, post-translationally altered, and able to build trimeric complexes with T-Ag and p53 as well as complexes with free p53 thereby inhibiting proteasomal degradation of p53 (249).

Abnormalities in p53 are also related to phenotypes of premature aging. Recently, a mechanistic connection between the proteasome activator REGγ, CK1δ, and p53 has been demonstrated using a mouse model for premature aging. In this pathway, CK1δ is degraded after direct binding to REGγ. Subsequently, degradation of MDM2 is disturbed due to the lack of CK1δ and p53 levels decrease. These findings provide new insights to the conversely discussed anti- and pro-aging effects of p53 (250).

Obviously, CK1 family members are involved in p53-related signal transduction in response to cellular stress conditions in numerous ways (Figure 4). However, in most cases upstream regulators and the mechanism of CK1 activity regulation in these pathways still remain unknown. Another component in DNA damage-initiated signal transduction, being targeted by CK1 isoforms, is the protein phosphatase Cdc25A (cell division cycle 25A). Activation of cyclin-dependent kinases (Cdks) by dephosphorylation mediated by Cdc25 is required for cell cycle progression from G1 to S phase (251). Among phosphorylation by other cellular kinases, site-specific phosphorylation of Cdc25A by CK1α and ε at residues Ser-79 and Ser-82 targets Cdc25A for degradation via the ubiquitin-proteasome pathway (141, 143). This CK1-regulated degradation of Cdc25A supports DNA damage-induced cell cycle arrest, which is mediated via inhibition of Cdks by p53 and p21 (252). Since CK1 isoforms are involved in both, the degradation of Cdc25A as well as of p53, CK1 family members might act in a synergistic way to initiate cell cycle arrest.

In addition, CK1γ1 is related to DNA damage signaling by catalyzing the phosphorylation of claspin, an adaptor protein critically involved in ATR-mediated activation of Chk1. In this context, CK1γ1-mediated phosphorylation of claspin enhances its binding to Chk1 (162). Chk1 in turn has been identified as a cellular kinase phosphorylating CK1δ leading to decreased CK1δ-specific activity (38). The significance of this observation for the p53/MDM2/CK1-signaling network remains to be determined. However, given the information that Chk1 is down-regulated by p53 activation the Chk1/CK1δ/p53-interconnection might be involved in fine-tuning the negative regulatory effect of p53 on Chk1 (253).

In hematopoietic cells, the physical interaction of CK1ε with PTEN (phosphatase and tensin homolog) has been proposed to modulate cell survival. Normally, constitutively active Akt kinase or Akt activated by the upstream phosphatidylinositol 3-kinase (PI3K) leads to the inhibition of p53 and p53-induced apoptosis, thereby providing a resistance mechanism for genotoxic stress (254, 255). However, in case PTEN is stimulated as shown for the interaction of PTEN with CK1ε, PI3K-mediated Akt activation is inhibited. Subsequent inhibition of p53 via active Akt is circumvented and the cells’ sensitivity toward genotoxic stress can be restored (256).

In the context of Akt signaling, CK1α was reported to affect DEPTOR, an inhibitor of the mTOR (mammalian Target of Rapamycin) kinase, which regulates cell growth, proliferation, and survival [reviewed in Sarbassov et al. (257)]. Phosphorylation of DEPTOR by CK1α leads to βTrCP-mediated proteasomal degradation of DEPTOR resulting in activation of mTOR signaling, which is consistent with DEPTOR down-regulation and mTOR activation found in many cancers (258). Therefore, CK1α might provide a therapeutic target for the treatment of cancers characterized by low DEPTOR levels and activation of mTOR signaling, leading to increasing DEPTOR levels, and inhibition of mTOR signaling. Paradoxically, DEPTOR is overexpressed in multiple myeloma, which is necessary for PI3K-mediated activation of Akt and thereby inhibition of p53 and p53-induced apoptosis (259, 260).

Under conditions of genotoxic stress rapid changes in connexin-43 (Cx43) leading to alterations in gap junction-dependent intercellular communication have been observed in corneal endothelial cells associated with stabilization of gap junction communication (261). Earlier reports already showed phosphorylation of Cx43 by CK1δ, which stimulates the incorporation of Cx43 into gap junction plaques and which therefore most likely also takes part in long term cellular adaptations in response to genotoxic stress (76).

Further DNA-associated proteins being modulated by CK1 isoforms are topoisomerases. For topoisomerase IIα, phosphorylation of Ser-1106 by CK1δ and ε has been demonstrated (98). This phosphorylation event is linked to enhanced DNA-cleavage activity of topoisomerase IIα via the stabilization of topoisomerase-DNA cleavable complexes after etoposide treatment (98).

### CK1 in circadian rhythm and its connections to stress response

**Figure 5:**
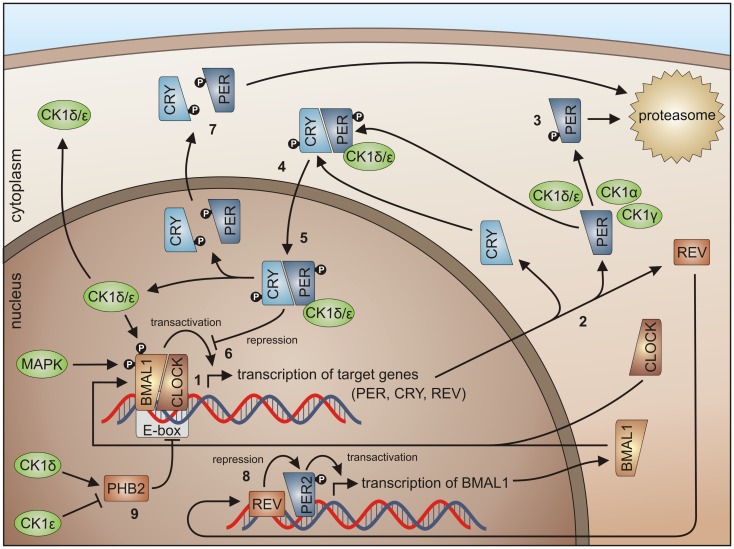
**CK1 in circadian rhythm regulation**. By binding of the BMAL1/CLOCK heterodimer to the E-box, transcription of E-box-containing genes is initiated (1) (here shown for PER1-3, CRY1 and 2, and REV-ERBα), the transcripts are translated in the cytoplasm (2). Degradation of cytoplasmic PERs is triggered mainly by CK1δ and ε, but also by isoforms α and γ (3), while PER degradation is inhibited by binding of CRYs to PERs (4). Subsequently, complexes of CRY/PER and CK1δ/ε translocate to the nucleus (5). In the nucleus the CRY/PER complex represses the transcriptional activation of BMAL1/CLOCK target genes (6). CRYs and PERs finally shuttle back to the cytoplasm for proteasomal degradation (7). Repression of BMAL1 expression by REV-ERBα represents a second negative feedback loop (8). Together, these feedback loops are able to generate cyclic expression of BMAL1 and E-box-containing genes [for review see Knippschild et al. (219) and Cheong and Virshup (195)]. CK1δ and ε differentially effect expression of the period length modulator PHB2. Whereas CK1δ is able to promote PHB2 transcription, its expression is reduced by CK1ε (9) (8, 263).

In almost every higher organism, an autonomous timer is known and referred to as the circadian clock. This timer consists of a signal transduction pathway to integrate external signals for time adjustment, a molecular oscillator generating the circadian signal, and a signal transduction pathway controlling the circadian periodicity of certain biological processes. Therefore, circadian proteins are closely connected to key regulators of the cell cycle, oxidative stress, and carcinogenesis. Basically, in the mammalian circadian clock the positive regulators CLOCK and brain and muscle ARNT-like protein (BMAL1) as well as the negative regulators PERIOD (PER) and CRYPTOCHROME (CRY) form an oscillating system controlling their own expression levels (Figure 5) [reviewed in more detail in Kelleher et al. (262)].

Linking circadian rhythm to cell cycle control, the heterodimer CLOCK/BMAL1 transcriptionally controls the expression of cell cycle regulators. PER1 interacts with ATM and Chk2 (264), whereas TIM, the mammalian homolog of *Drosophila* timeless protein, interacts with Chk1, ATR, and the ATR-related protein ATRIP (265). Furthermore, BMAL1 was identified to be necessary for p53-dependent growth arrest in response to DNA damage (266). Within the metabolism of reactive oxygen species (ROS) circadian proteins also seem to be involved, since the circadian clock could offer reliable control of daily variation in antioxidant response necessary to counteract increased oxidative stress. This connection is reasonable and important as oxidative stress is linked to the pathogenesis of cardiovascular diseases, atherosclerosis, and cancer (267). As an example, BMAL1 deficiency leads to chronic oxidative stress and an accelerated aging phenotype in mice (268). Conversely, activity of the circadian clock itself can be regulated by components of ROS metabolism (269). Finally, the circadian clock is also linked to the development of cancer. For PER2 mutant mice increased formation of radiation-induced lymphomas was reported and the frequency (FRQ) of intestinal and colonic polyps was increased in APC^min/+^PER2^m/m^ mice compared to APC^min/+^ littermates (270).

In order to control the circadian rhythm involved, regulating components are subject to post-translational modifications like reversible phosphorylation (271). In general, CK1 isoforms δ and ε are able to phosphorylate and regulate the clock proteins BMAL1 and CRY and can modulate the expression of the period length modulator prohibitin 2 (PHB2) (114, 263). CK1δ is seen as an important regulator in circadian rhythm but also the involvement of other CK1 isoforms has been detected. CK1δ and ε are able to influence the length of the circadian period by regulating the stability and subcellular localization of PER (Figure 5) (149, 163, 272, 273). Phosphorylation of PER1 by CK1ε masks the nuclear localization signal of PER1 by conformational changes and marks PER for proteasomal degradation (149). CK1δ and ε interact with PER/CRY complexes thereby promoting nuclear localization of PER/CRY complexes (149, 274). In a high-throughput compound screening also CK1α was found to stimulate the degradation of PER1. In this screen, the protein kinases CK1α, CK1δ, and ERK2 were identified as targets for the compound *longdaysin*. However, CK1α binding affinity to PER1 is much weaker than for CK1δ or ε (275). The same is true for CK1γ (276). Thus, CK1δ and ε can be regarded as redundant for PER phosphorylation and essential for nuclear accumulation of PER (277). Inhibition of CK1δ and ε by the pan-CK1δ/ε inhibitor PF-670462 led to remarkably lengthened circadian rhythms (*in vivo* locomotor activity) and molecular oscillations (*in vitro* in the suprachiasmatic nucleus and peripheral tissue slices). These observations could not be made using the CK1ε-specific inhibitor PF-4800567 (278). PF-4800567 effectively blocked CK1ε-mediated nuclear localization of PER3 and degradation of PER2 but only showed minimal effect on the circadian clock in cycling Rat1 fibroblasts (273). The CK1ε *tau* mutation, however, which was discovered in the Syrian hamster as the first mammalian circadian mutation, was characterized as a gain of function mutation resulting in clock acceleration. In mice expressing the CK1ε *tau* mutation increased phosphorylation of PER1 and 2 can be detected leading to increased degradation of nuclear and cytoplasmic PER and acceleration of the mammalian clock (279, 280). Um and colleagues discovered, that the circadian period of Rat1 fibroblasts treated with the diabetes drug metformin was shortened by 1 h. By metformin treatment, AMP-activated kinase (AMPK) is activated, which phosphorylates CK1ε at Ser-389 leading to increased activity of CK1ε and subsequent degradation of Per2 (281). A higher level regulator of CK1ε activity in circadian rhythm is protein phosphatase 5 (PP5), which can raise the activity of CK1ε by dephosphorylation. As a consequence, phosphorylation by CK1ε and subsequent degradation of PER is also increased (282). Recently, CK1δ (but not CK1ε) has been shown to be crucial for the circadian timing mechanism in zebrafish (283).

Presented observations point to PER proteins as multikinase targets, which can be multiply phosphorylated and thereby regulated. Herein, the balance between phosphorylation and dephosphorylation by phosphatases is of certain importance. In cells deficient for CK1δ and ε, phosphorylation of PER is disturbed and PER proteins remain cytoplasmic. In case protein phosphatase 1 (PP1) is disrupted, phosphorylation of PER is accelerated. This effect is specific to PP1 and in contrast to previous *Drosophila* studies cannot be observed for PP2A (276).

### CK1-signaling in apoptotic pathways

For several CK1 isoforms, an involvement in the regulation of apoptotic signal transduction has been described. CK1α, δ, and ε are components of Fas-mediated apoptosis and induce an activation of initiator caspase 8. Here the pro-apoptotic protein Bid, which belongs to the Bcl-2 family, is of major interest. Amino acids Ser-64 and Ser-66 of Bid are supposed to be major targets for CK1-mediated phosphorylation while Thr-58 is targeted by CK2. Only unphosphorylated Bid can be processed by caspase 8-mediated proteolysis and can participate in cytochrome c-mediated apoptosis. Accordingly, inhibition of CK1 and CK2 induces accelerated Fas-triggered apoptosis by blocking inhibitory phosphorylation of Bid. Vice versa an overexpression of CK1ε and CK2 leads to a decreased number of apoptotic cells due to increased phosphorylation of Bid, blocking its caspase 8-mediated processing. Therefore, phosphorylation of Bid by CK1δ and ε and CK2 can inhibit Fas-mediated apoptosis (151).

Moreover, CK1 (isolated from pig spleen) can phosphorylate the p75 neurotrophin receptor, thereby negatively regulating p75-mediated apoptosis (284). CK1α is involved in apoptosis by interaction and phosphorylation of retinoid X receptor (RXR), a class of retinoic acid receptors regulating cell survival by building heterodimers with NGF1B (nerve growth factor 1B), IGFBP-3 (insulin-like growth factor binding protein 3), and β-catenin. In this case, CK1 activity inhibits the induction of apoptosis by RXR agonists (85, 285–287).

Furthermore, CK1α is able to phosphorylate FADD (Fas-associated protein with death domain) at Ser-194 *in vitro* as well as *in vivo* and is supposed to be involved in regulating non-apoptotic functions of FADD like cell cycle interaction, sensitivity toward chemotherapeutics, and nuclear localization (176, 288). In erythrocytes, CK1α modulates cytosolic calcium activity and thereby regulates programed cell death (289).

## Participation of CK1 in the Development of Cancer

During animal development, a precise coordination of cell patterning events is required to ensure appropriate organ architecture and size. Several developmental pathways control growth, proliferation, and apoptosis by strict regulation, which can result in pathological conditions when dysregulated. The Wnt (Wingless/Int-1), Hh (Hedgehog), and Hippo signaling pathways are important in tissue development, growth, and homeostasis (290–293). Aberrant activation of these pathways as well as mutations of components of these pathways has been linked to various cancer entities (294–298). Due to the contribution of CK1 family members in pathways associated with growth and development, the following sections concentrate on the current knowledge of CK1 participation and regulation in the Wnt, Hh, and Hippo signaling pathways.

### CK1 and the Wnt pathway

Components of the Wnt-signaling pathway are involved in many developmental processes including dorsal axis formation, tissue patterning, and establishment of cell polarity (299–302). In addition, Wnt/β-catenin-mediated signaling plays an important regulatory role in cell proliferation processes in both, embryonic and mature organisms. Mutations in Wnt pathway components have been found in various human cancers, including cancers of the skin, liver, brain, and colon (291, 303–312).

**Figure 6:**
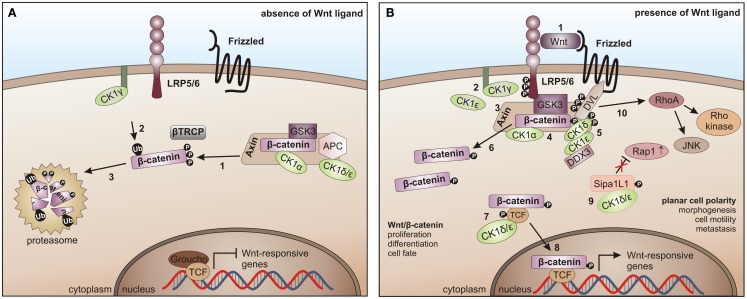
**CK1 in Wnt-signaling**. **(A)** In the absence of the Wnt ligand, β-catenin is progressively phosphorylated by CK1α and GSK3 (1), recruited to β-TrCP for ubiquitination (2), and thereby primed for proteasome- dependent degradation (3). **(B)** After binding of Wnt to Frizzled and LRP5/6 (1), LRP5/6 is phosphorylated by CK1γ (positive regulation) and CK1ε (negative regulation) (2). It then recruits Axin and the β-catenin destruction complex to the membrane and inhibits GSK3 (3, 4). Wnt-activated CK1δ and ε phosphorylate Disheveled (DVL) and Axin (5), induce a conformational change in the β-catenin destruction complex and initiate the dissociation of various components (6). CK1ε cooperates with DDX3 in phosphorylating DVL (7). Also, TCF3 can be phosphorylated by CK1δ and ε thereby increasing its binding to β-catenin followed by the nuclear translocation of TCF3/β- catenin (8). The non-canonical Wnt pathway is positively regulated by CK1δ- and ε-dependent release of Rap1 from Sipa1L1 inhibition (9). The Rho/JNK signaling cascade is activated after phosphorylation of DVL (10) [adapted from Cheong and Virshup (195)].

In the canonical Wnt/β-catenin signaling pathway, all CK1 family members are involved. However, this involvement is quite complex. So far, positive as well as negative regulatory functions have been described. In absence of the Wnt ligand CK1α interacts with and phosphorylates Axin, adenomatous polyposis coli (APC), and β-catenin (at Ser-45), thereby priming β-catenin for further phosphorylation by GSK3β and subsequent degradation (195, 313) (Figure 6A). After binding of Wnt ligand to Frizzled (Fzd) the Wnt co-receptor LRP5/6 is phosphorylated either by membrane-bound CK1γ (positive regulation) (86) or by CK1ε (negative regulation) (314). Phosphorylated LRP5/6 then recruits Axin and the β-catenin destruction complex to the membrane and inhibits GSK3β. Wnt-activated CK1δ and CK1ε phosphorylate Axin as well as the scaffold protein DVL at multiple sites and can introduce a conformational change to the β-catenin destruction complex followed by dissociation of several components, thereby preventing β-catenin from being phosphorylated and degraded (148, 195). Recently, RNA helicase DDX3 was identified as a regulatory subunit of CK1ε in Wnt-signaling. Wnt-activation promotes recruitment of DDX3 to CK1ε and binding directly stimulates kinase activity, promoting phosphorylation of DVL, finally leading to stabilization of β-catenin (103). Accumulated β-catenin then translocates to the nucleus to activate the expression of TCF/LEF (T cell factor/lymphoid enhancing factor)-triggered target genes (Figure 6B) (291). CK1ε is also involved in the formation of an active transcription complex by phosphorylating TCF3 thereby mediating its activation and binding to β-catenin (113).

CK1ε is associated with a positive regulatory function by joining the Wnt multi-protein complex to phosphorylate DVL, which in turn gets activated and inhibits GSK3β, finally resulting in stabilization of β-catenin (148).

Signaling in the non-canonical Wnt pathway is positively regulated by CK1δ and ε, which release Rap1 from Sipa1L1 inhibition. Subsequent to phosphorylation of DVL the Rho/JNK signaling cascade can be activated (Figure 6B) [reviewed in Cheong and Virshup (195)].

### CK1 in the hedgehog pathway

The Hh signaling pathway regulates a variety of processes during embryonic development such as differentiation, proliferation, and organogenesis (290). In the adult organism, Hh signaling is significantly reduced but plays a critical role in regulating epithelial maintenance and regeneration of organs, which undergo constant renewal; among them, epithelia of internal organs and brain (315). Therefore, mutations or dysregulation of components of this pathway are associated with tumorigenesis and cancer development, including basal cell carcinomas, medulloblastomas, gliomas, gastrointestinal tumors, prostate cancer, and hematological malignancies (315–318).

**Figure 7:**
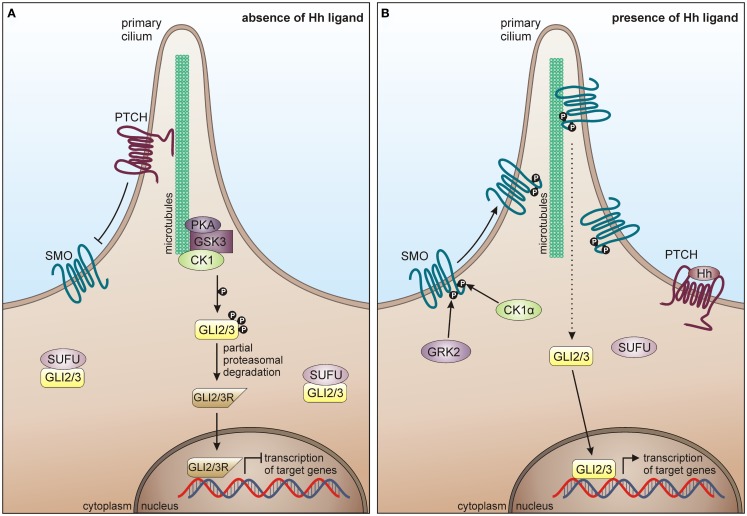
**CK1 in Hh signaling**. **(A)** In absence of Hh ligand, PTCH localizes in the cilium and inhibits surface trafficking and cilia localization of SMO. GLI proteins are phosphorylated by PKA, GSK3β, and CK1, which lead to proteasome-dependent cleavage of GLI into a N-terminally truncated form, generating the repressor forms GLI2R and GLI3R. GLI2/3R translocate to the nucleus and inhibit translation of Hh target genes. Furthermore, SUFU prevents GLI from activating Hh target genes, by binding it in the cytoplasm and the nucleus. **(B)** In response to Hh, SMO is activated by GRK2 and CK1α-dependent phosphorylation and enters the primary cilium. Activated SMO orchestrates a signaling cascade, eventually resulting in the dissociation of the SUFU-GLI complex and the translocation of full-length GLI2/3 to the nucleus, where Hh target gene expression is induced.

In mammals, major components of the Hh pathway are represented by the three Hh homologous ligands Sonic hedgehog (Shh), Indian hedgehog (Ihh), and Desert hedgehog (Dhh), the negative regulatory 12-pass membrane receptor Patched (PTCH), the positive regulatory 7-pass membrane protein smoothened (SMO), the glioma-associated oncogene (GLI) transcription factors GLI1, GLI2, GLI3, a multi-protein complex consisting of intraflagellar transport proteins, protein kinase A (PKA), GSK3, CK1, and suppressor of fused (SUFU) (319). In absence of Hh ligands, PTCH inhibits the localization of SMO to the cilia cell surface and represses SMO activity, thereby suppressing signal transmission via the GLI transcription factors into the nucleus. PKA, GSK3β, and CK1 phosphorylate the GLI transcription factors leading to their proteolytic processing into the repressor forms, which cannot activate target gene transcription (Figure 7A) (320, 321). Hh signal transduction is initiated upon binding of a Hh ligand to PTCH, thereby releasing SMO from PTCH-mediated inhibition, leading to its accumulation on cilia cell surface and consequent activation and release of the GLI transcription factors from the multi-protein complex. Activated GLIs then translocate to the nucleus, where they induce transcription of Hh target genes (Figure 7B) (319).

In 2002, Price and Kalderon postulated a negative regulatory role of CK1 in Hh signaling in *Drosophila melanogaster* (322). They demonstrated that CK1δ- and GSK3-mediated phosphorylation of Ci-155 (full-length Cubitus interruptus, the *Drosophila* homolog of GLI2 and GLI3) at PKA primed sites is required for the partial proteolysis of the transcription factors, thereby preventing Hh target gene transcription [reviewed in Price (323)]. The PKA, GSK3, and CK1 sites are conserved in Ci, GLI2, and GLI3, which are all similarly processed and may play similar roles in *Drosophila* and vertebrates (324–327). Furthermore, Wang and Li demonstrated, that CK1 and GSK3 phosphorylation sites are needed to process GLI3 (327). CK1 has also been implicated in positive regulation of SMO. Chen and co-workers demonstrated that mammalian SMO is activated via multiple phosphorylation events mediated by CK1α and G protein coupled receptor kinase 2 (GRK2), thereby inducing its cilia accumulation and active conformation (328).

### CK1 in the Hippo pathway

During development, the evolutionary conserved Hippo pathway contributes to several processes, which restrict organ size by controlling cell proliferation and apoptosis [reviewed in Zhao et al. (124)]. Consequently, pathway deregulation can trigger tumorigenesis and occurs in a broad range of human cancers. Abnormal Hippo activity is associated with cancer cell proliferation, enhanced cell survival, and maintenance of a stem cell phenotype [reviewed in Harvey et al. (329)].

**Figure 8:**
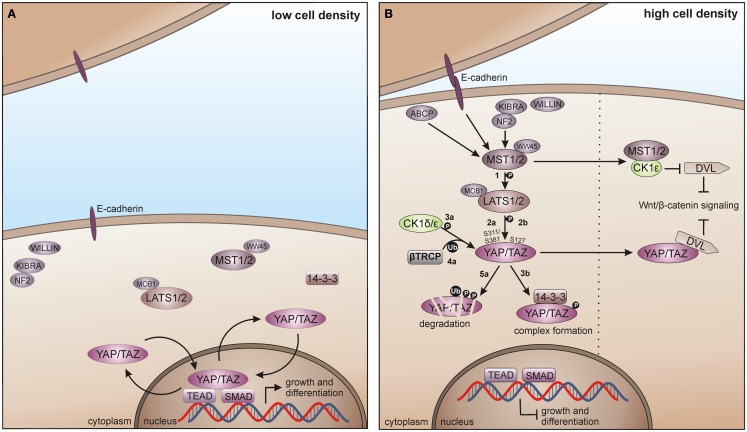
**CK1 in Hippo signaling in vertebrates**. **(A)** In absence of growth suppressive signals YAP/TAZ promotes tissue growth and differentiation by regulating the activity of different transcription factors in the nucleus, including SMADs and TEADs. **(B)** Cell-density activated pathway regulation is controlled by multiple upstream branches by activating the core kinase cassette that represses YAP/TAZ driven gene transcription, either by degradation of TAP/TAZ or by forming physical complexes, preventing its nuclear access. Initially, MST1/2 is activated by various components and phosphorylates LATS1/2 (1), which in turn phosphorylates TAP/TAZ on Ser-311 or Ser-381 (2a). This phosphorylation primes YAP/TAZ for further phosphorylation by CK1δ/ε (3a) and consequent recruitment of and ubiquitination by β-TrCP (4a), priming YAP/TAZ for degradation (5a). However, LATS1/2 driven phosphorylation of TAP/TAZ on Ser-127 (2b) leads to the formation of 14-3-3-YAP/TAZ complexes, which accumulate in the cytoplasm preventing YAP/TAZ access to the nucleus (3b). Hippo pathway regulates Wnt/β-catenin signaling by inhibition of DVL, either by MST1/2-mediated prevention of CK1ε-dependent phosphorylation of DVL, or by direct inhibition of DVL by YAP/TAZ. ABCP: apicobasal cell polarity protein.

The mammalian Hippo pathway is initiated by various growth suppressive signals like cell contact inhibition. The upstream kinases mammalian STE20-like protein kinase 1/2 (MST1/2), together with the scaffold proteins vertebrate homolog of *Drosophila* Salvador (WW45) and MOB kinase activator 1A/B (MOB1A/B) phosphorylate the large tumor suppressor 1 and 2 (LATS1/2). LATS1/2-dependent phosphorylation of the transcriptional co-activator Yes-associated protein (YAP) and its paralog Tafazzin (TAZ) then leads to YAP/TAZ inhibition by spatial separation from its nuclear target transcription factors TEAD (TEA domain) and SMAD (SMA/mothers against decapentaplegic) and additionally by phosphodegron-mediated degradation, preventing Hippo target gene transcription (Figure 8A) (330, 331).

Zhao and co-workers as well as Liu and co-workers identified CK1δ and ε as new temporal regulators of the Hippo pathway. YAP is phosphorylated by LATS on Ser-381 and this phosphorylation provides the priming signal for CK1δ or ε to phosphorylate a phosphodegron in YAP, which in turn recruits β-TrCP leading to YAP ubiquitination and degradation (124). Furthermore, TAZ phosphorylation at Ser-311 by LATS also leads to subsequent CK1ε-mediated phosphorylation of a phosphodegron in TAZ and consequently to its degradation (123). Xu and co-workers recently postulated the interaction of the Hippo and Wnt pathway via CK1ε. Herein, the Hippo upstream kinase MST1 is able to suppress the Wnt/β-catenin pathway by directly binding CK1ε, thereby preventing phosphorylation of DVL (Figure 8B) (332).

### CK1-related tumorigenic functions

The important role of CK1 family members within various signaling pathways is furthermore supported by reports linking CK1 isoforms to modulation of key regulatory proteins such as p53, MDM2, and β-catenin, which act as signal integration molecules in stress situations and generally can be seen as a key regulatory connection to tumorigenesis [for more detailed review see Knippschild et al. (219, 333), and Cheong and Virshup (195)]. Considering the importance of signals mediated by CK1δ and ε to finally ensure genome stability, it is obvious that mutations leading to changes in the activity or expression levels of CK1 isoforms or mutations of CK1-specific target sites in its substrates can contribute to the development of cancer (Table 2). Foldynová-Trantírková and co-workers provided evidence that mutations in CK1ε, which are frequently found in breast cancer, lead to loss of function in the Wnt/β-catenin pathway but result in activation of the Wnt/Rac1/JNK and Wnt/Ca^2+^ pathway, consequently leading to increased migratory capacity and decreased cell-adhesion (334). A mutation within the C-terminal region of CK1δ detected in an adenomatous colorectal polyp leads to a higher oncogenic potential and promotes the development of adenomas in the intestinal mucosa (335). Furthermore, conditional knock-out of CK1α in the intestinal epithelium leads to activation of p53 and Wnt-signaling, while in p53 deficient gut, loss of heterozygosity of the CK1α gene causes a highly invasive carcinoma, indicating that CK1α acts as a tumor suppressor when p53 is inactivated (336).

**Table 2:** **CK1 isoforms in different tumor entities**.

Isoform	Characteristic feature	Tumor entity	Reference
CK1α	Low/absent expression	Primary/metastatic melanoma, lymphomas, ovarian, breast, and colon carcinomas	(337)
CK1γ3	Altered activity/expression	Renal cell carcinoma	(338)
CK1δ	Increased expression levels	Choriocarcinomas	(222)
CK1δ	Reduced immunostaining	Poorly differentiated breast carcinomas and DCIS	(219)
CK1δ/ε	Elevated protein levels	High-grade ductal pancreatic carcinomas	(339)
CK1ε	Reduced expression levels	Pancreatic ductal adenocarcinoma	(340)
CK1ε	Increased immunoreactivity	Mammary DCIS	(184, 341)
	Decreased immunoreactivity	Invasive mammary carcinoma	
CK1ε	Overexpression	Breast cancer	(342)
CK1ε	High gene expression	Adenoid cystic carcinoma of the salivary gland	(343)
CK1ε	Overexpression	Epithelial ovarian cancer	(344)
CK1ε	Overexpression	Tumors of brain, head and neck, renal, bladder, lung, prostate, salivary gland, leukemia, melanoma, and seminoma	(345)
CK1ε	Overexpression	MYC-driven cancers (neuroblastoma, colon, lung, and breast cancer)	(346)
CK1ε	Loss of cytoplasmic expression	Poor prognosis in oral cancer patients	(347)

In 1981, Elias and co-workers already reported an increased nuclear CK1 kinase activity in AML patients (186). Until now, several reports link altered CK1 expression and/or activity to cancer. Reduced CK1α protein and mRNA expression levels in primary melanomas and melanoma metastases compared to benign melanocytic lesions or early-stage melanomas have been detected. In the same study, reduced CK1α expression was also observed in lymphomas, ovarian, breast, and colon carcinomas, compared with the respective benign tissue (337). In renal cell carcinoma elevated CK1γ3 expression and activity levels have been described (338), whereas in choriocarcinomas strong expression levels of CK1δ were detected (222). Changes in the immunoreactivity of CK1δ have been observed in breast carcinomas, depending on the grade of tumor differentiation. High-grade ductal carcinomas *in situ* (DCIS) as well as invasive poorly differentiated carcinomas show reduced CK1δ immunostaining, whereas well differentiated carcinomas and low grade DCIS show strong staining of tumor cells (219). Regarding CK1ε, Fuja and co-workers observed similar correlations between tumor differentiation and immunohistological staining (341). Expression of CK1ε is also down-regulated in mammary cancers in SV40-transgenic mice expressing SV40 T-Ag (184). A recent study suggests that CK1ε is overexpressed in breast tumors and acts as a pivotal regulator of mRNA translation and cell proliferation. CK1ε phosphorylates the negative-acting factor 4E-BP1 (eukaryotic translation initiation factor 4E binding protein 1), thereby preventing its inhibitory function on the translation initiation complex elF4E (eukaryotic initiation factor 4E) and consequently leading to dysregulated mRNA translation and breast cancer cell growth (342). Elevated protein levels of CK1δ and ε were also observed in single tumor cells of grade 3 tumors of ductal pancreatic carcinomas and inhibition of CK1δ and ε by the CK1-specific inhibitor IC261 reduced pancreatic tumor cell growth in xenografts (339). In contrast, Relles and co-workers detected reduced expression levels in pancreatic ductal adenocarcinomas (340). CK1ε expression is increased in adenoid cystic carcinomas of the salivary gland (343), in epithelial ovarian cancer (344), in tumors of brain, head and neck, renal, bladder, lung, prostate, and salivary gland, in leukemia, melanoma, and seminoma (345). Toyoshima and co-workers found that CK1ε expression is significantly correlated with MYCN amplification in neuroblastoma and poor prognosis. In addition, CK1ε expression has been associated with c-MYC in several other tumors such as colon, lung, and breast cancer (346). In a recent study, Lin and co-workers demonstrated that loss of cytoplasmatic CK1ε expression correlates with poor survival rates in oral squamous cell carcinoma (347). Järas and co-workers recently found that CK1α is essential for AML cell survival and treatment with the CK1-specific inhibitor D4476 results in highly selective killing of leukemia stem cells by reducing Rsp6 (radial spoke protein 6) phosphorylation and activation of p53 (Table 4) (348).

In summary, the data reported so far provide evidence that CK1 isoforms exhibit oncogenic features by promoting proliferation, genome instability, and inhibition of apoptotic processes. This assumption is also supported by the fact that CK1 isoforms are often overexpressed in tumors and that overexpression of CK1ε correlates with poor survival as shown for patients with ovarian cancer (344). However, this finding cannot be generalized and might depend on additional factors, as in the case of oral squamous cell carcinoma loss of CK1ε expression correlates with poor survival rates (347). In addition, the functions of CK1α in tumorigenesis are manifold making it difficult to classify it as oncogene or tumor suppressor. In AML CK1α seems to exhibit oncogenic features (348), whereas in intestinal epithelium loss of heterozygosity of the CK1α gene causes a highly invasive carcinoma, indicating that CK1α acts as a tumor suppressor when p53 is inactivated (336).

### CK1 in metastatic processes

In many cases, CK1 family members can also be involved in the regulation of metastatic processes. However, their potential to promote or suppress metastasis seems to depend not on the specific isoform but on their position in cellular signal transduction and the cellular context. Phosphorylation of nm23-H1 by CK1δ and ε has been shown to induce complex formation of nm23-H1 with a cellular partner called h-prune. Both proteins are linked to proliferative disorders and the nm23-H1-h-prune complex formation has even been proposed to positively influence cell motility (349). With this link of CK1 kinase activity to nm23-H1-h-prune complex formation an obvious role for CK1 in the mediation of metastasis has been established (153).

Quite recently, the stability of metastasis-related proteins has been shown to be regulated by CK1δ-mediated phosphorylation. First, the epigenetic sensor UHRF1 is critically involved in the maintenance of DNA methylation patterns during DNA replication and can be linked to carcinogenesis and metastasis if dysregulated (350, 351). Second, as mentioned before, proteasomal degradation of UHRF1 is regulated by CK1δ-mediated phosphorylation (104). Similar findings have been reported for metastasis suppressor 1 (MTSS1, also known as MIM, missing in metastasis), an anti-metastatic protein whose degradation also is triggered by CK1δ-mediated phosphorylation at Ser-322, thereby inducing its interaction with SCF/β-TrCP (77, 352).

Furthermore, current reports demonstrate the involvement of CK1α in regulating the stability of metastasis-associated factors. When cell motility is induced the Rap guanine exchange factor (RAPGEF2) is phosphorylated by IKKβ and CK1α, initiating SCF/β-TrCP-mediated degradation. RAPGEF2 degradation-failure leads to inhibition of hepatocyte growth factor (HGF)-induced cell migration and expression of non-degradable RAPGEF2 suppressed metastasis of human breast cancer cells (165).

In canonical Wnt-signaling, CK1α has been positioned to be a tumor suppressor and cancer cells may activate proliferative processes via the Wnt/β-catenin pathway by suppressing CK1α expression. In the absence of CK1α, p53 is critically involved in controlling invasiveness as shown in a model for colon cancer (336). Re-expression of CK1α in metastatic melanoma cells reduced growth *in vitro* and metastasis formation *in vivo* (337). Consistent with these findings phosphorylation of β-catenin at Ser-45 by CK1α via activation by Wnt-5a has been shown to increase complex formation of β-catenin with E-cadherin thereby maintaining intercellular adhesion. Loss of Wnt-5a is thought to be associated with initial metastatic de-adhesion events (353, 354). Conversely, E-cadherin-mediated cell–cell contacts can be negatively regulated by CK1ε-mediated phosphorylation of E-cadherin at Ser-846 (69). In this context also, the Zn-finger transcription factor Snail is important as it can promote epithelial to mesenchymal transition (EMT) by down-regulating E-cadherin expression (355). Herein, CK1ε primes Snail for GSK3β-mediated phosphorylation, which marks Snail for degradation. Therefore, loss of CK1ε kinase activity prevents GSK3β-mediated phosphorylation and degradation of Snail supporting EMT and metastatic processes (122).

### CK1-specific inhibitors

Due to the obvious involvement of CK1 isoforms in the pathogenesis of inflammatory and proliferative diseases and its contribution to the development of neuro-degenerative disorders, CK1 family members are attracting more and more attention as drug targets in regard to therapeutic applications. So far, several highly potent CK1-specific small molecule inhibitors have been identified (Table 3) and some have already been characterized for their therapeutic potential in animal models (Table 4). Most of these compounds are ATP-competitive type I inhibitors raising the problem of comparability of their effectiveness since their IC_50_ values have been determined at different ATP concentrations (see Table 3).

**Table 3:** **CK1-specific small molecule inhibitors**.

Inhibitor	Structure	IC_50_ (μM)	ATP (μM)	Reference
CKI-7	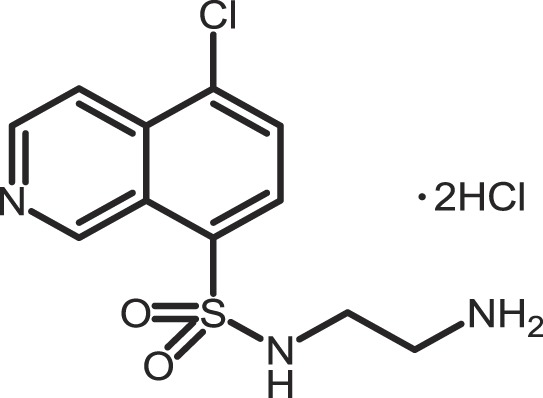	CK1: 6	100	(356, 357)
IC261	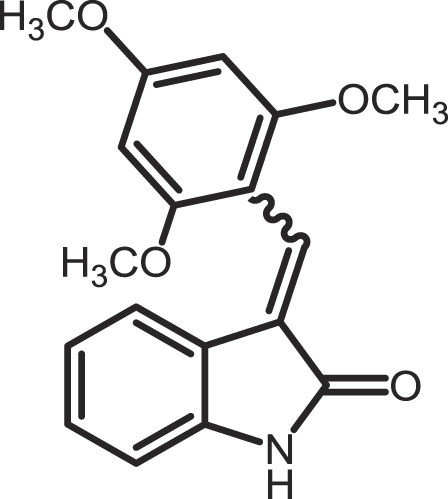	CK1δ/ε: 2.5	100	(357, 358)
D4476	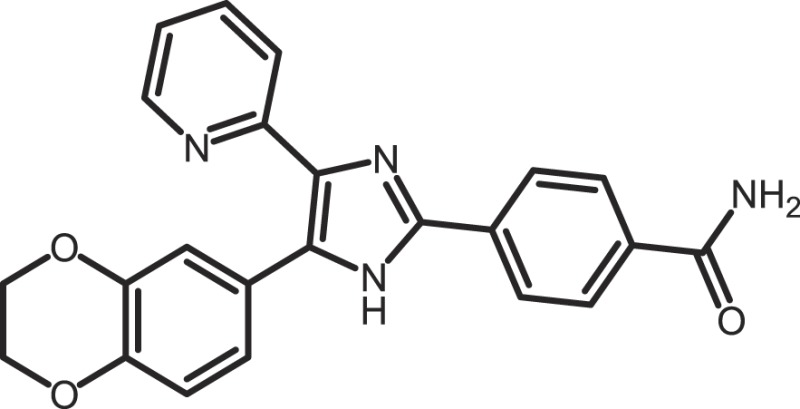	CK1δ: 0.3	100	(357)
Peifer-17	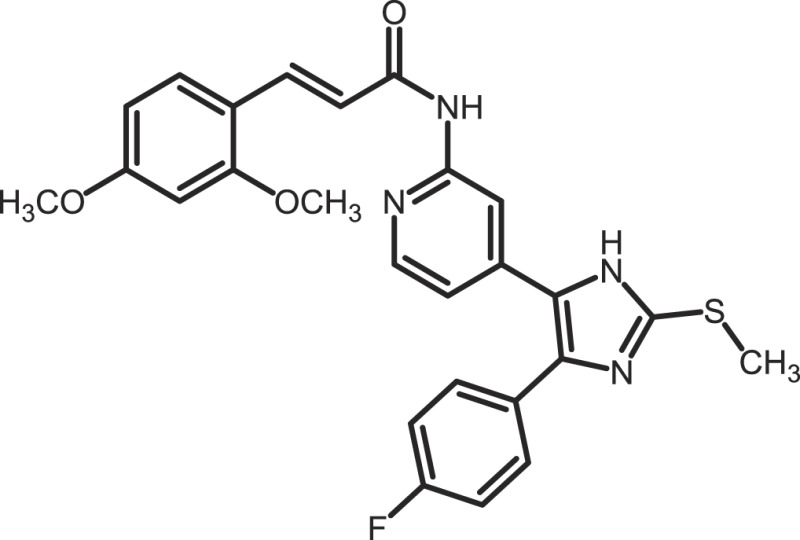	CK1δ: 0.005; CK1ε: 0.073	100	(31)
Peifer-18	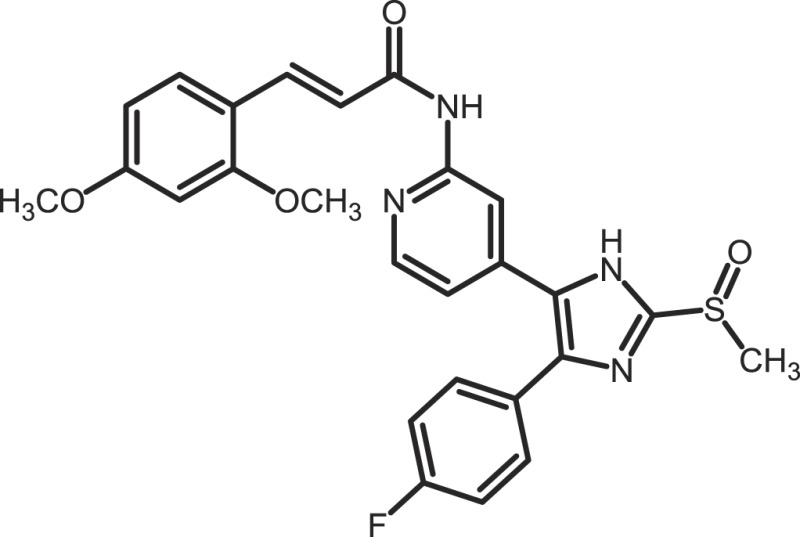	CK1δ: 0.011; CK1ε: 0.447	100	(31)
PF-670462	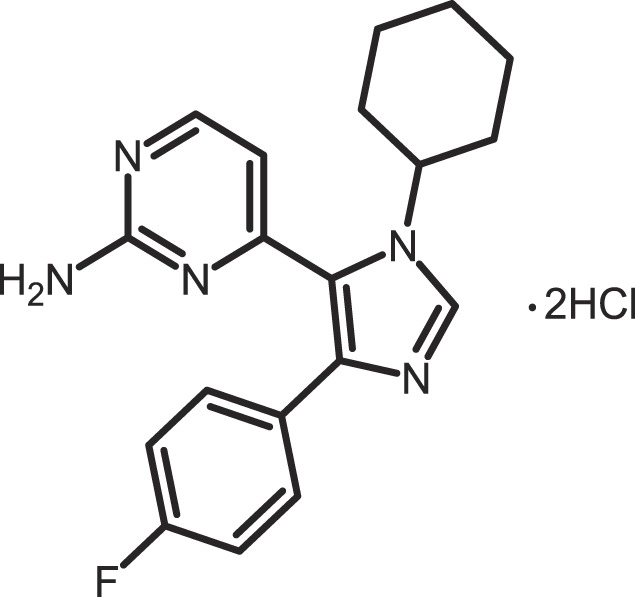	CK1δ: 0.013; CK1ε: 0.080	10	(273, 359)
PF-4800567	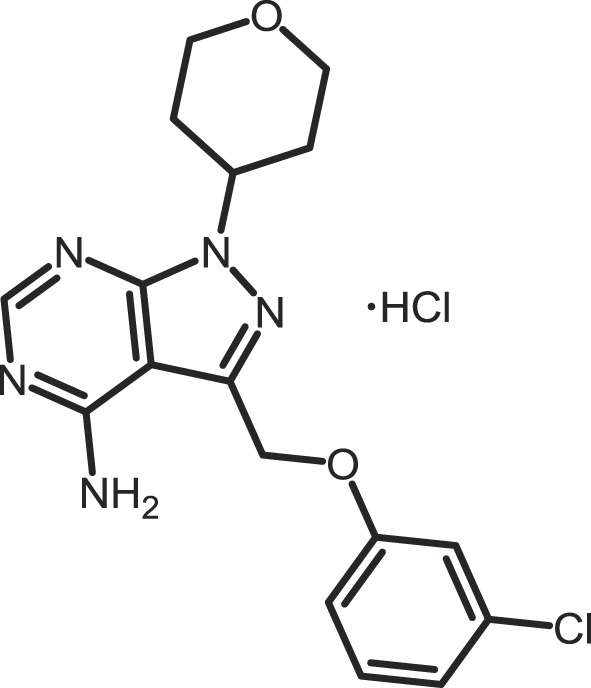	CK1δ: 0.711; CK1ε: 0.032	10	(273)
(R)-DRF053	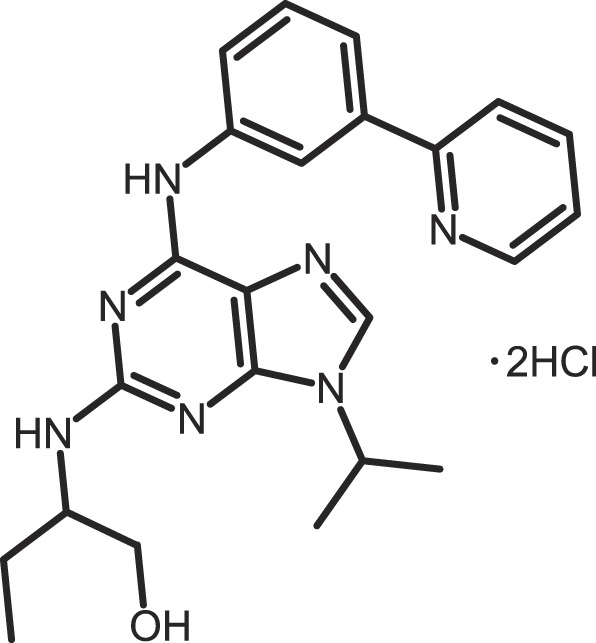	CK1δ/ε: 0.014	15	(360)
4,5,6,7-Tetrabromo-2-mercaptobenzimidazole	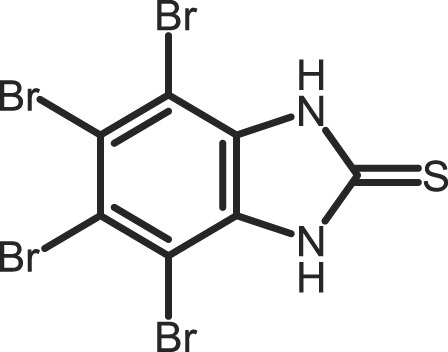	CK1: 2.2	20	(361)
1,4-Diaminoanthra-quinone	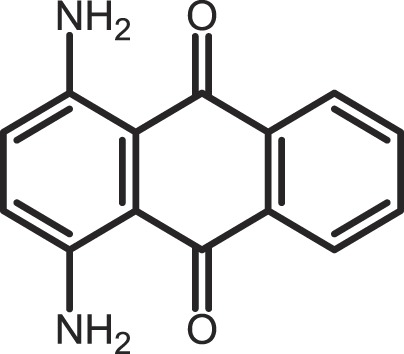	CK1δ: 0.3	Not reported	(362)
1-Hydroxy-4-aminoanthra-quinone	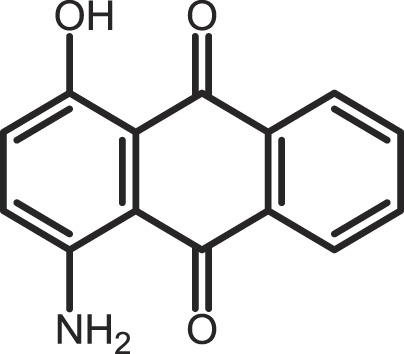	CK1δ: 0.6	Not reported	(362)
(−)-Matairenisol	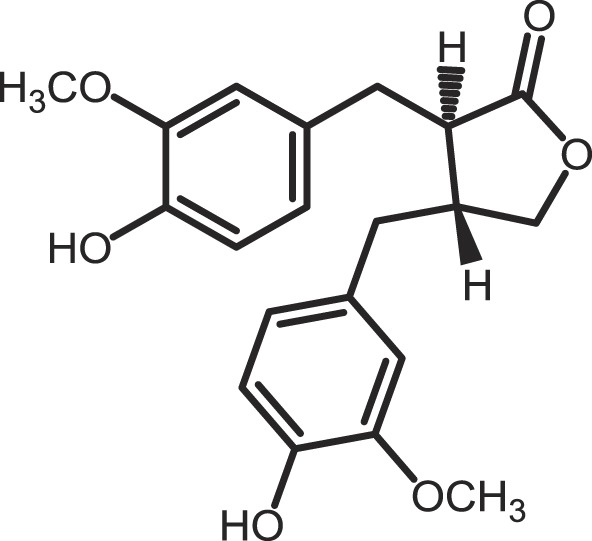	CK1: 10	10	(363)
Lamellarin 3	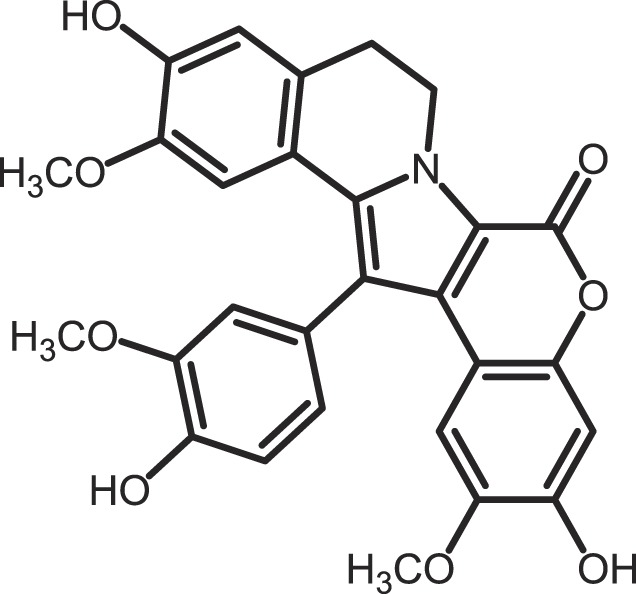	CK1δ/ε: 0.41	15	(364)
Lamellarin 6	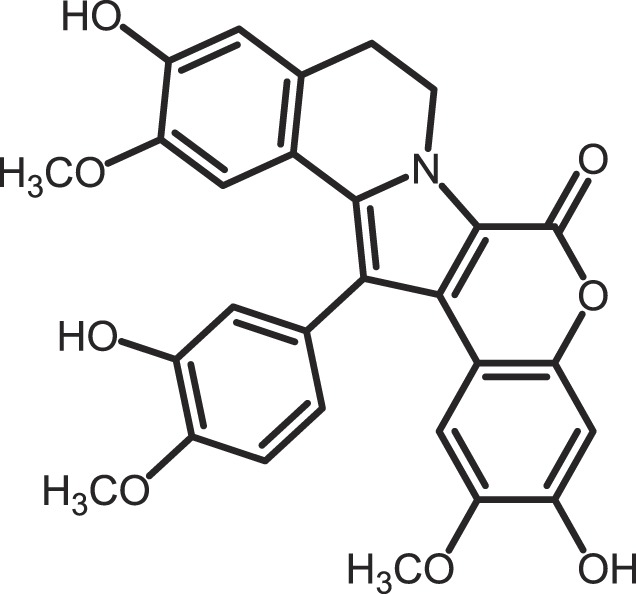	CK1δ/ε: 0.8	15	(364)
SB-202190	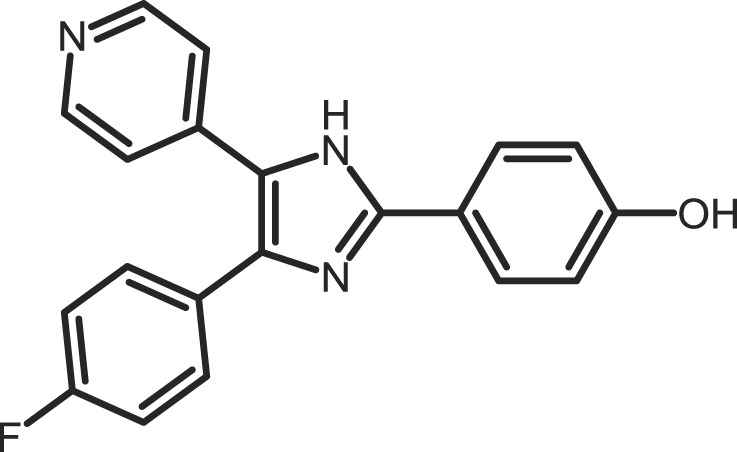	CK1δ: 0.6	50	(365)
SR-3029	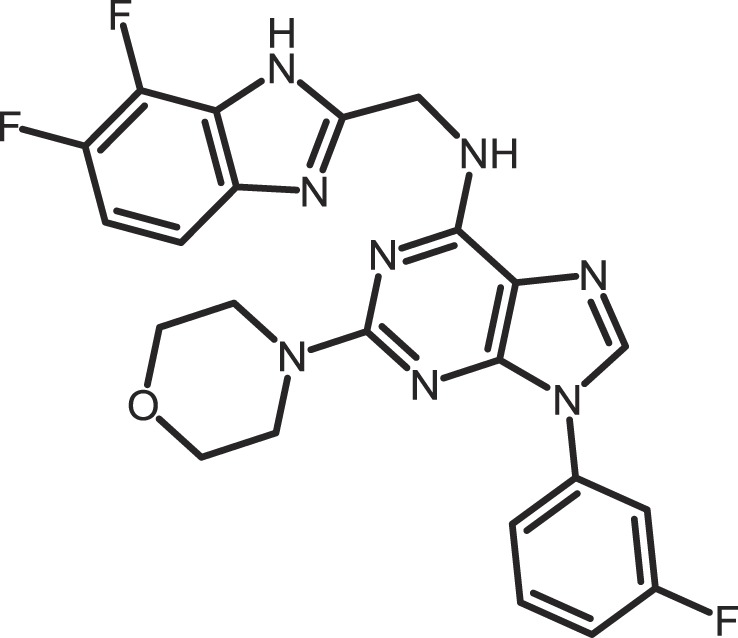	CK1δ: 0.044	10	(366)
SR-2890	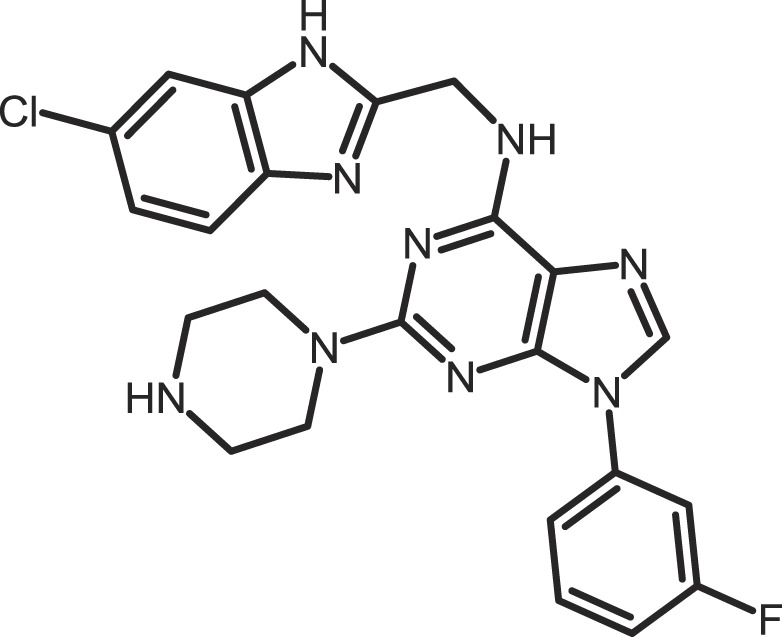	CK1δ: 0.004	10	(366)
Bischof-5	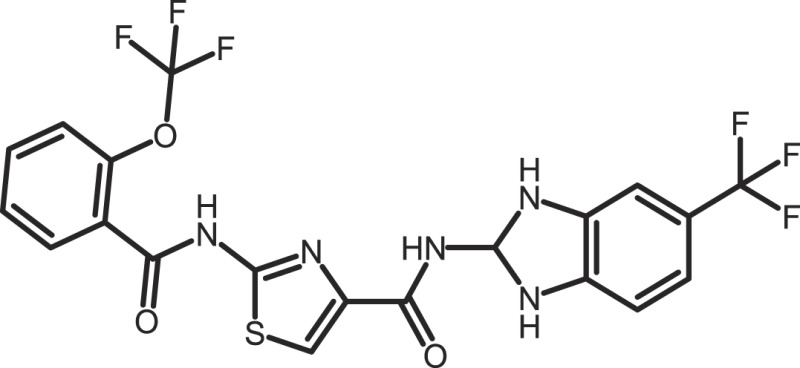	CK1δ: 0.04; CK1ε: 0.199	10	(367)
Bischof-6	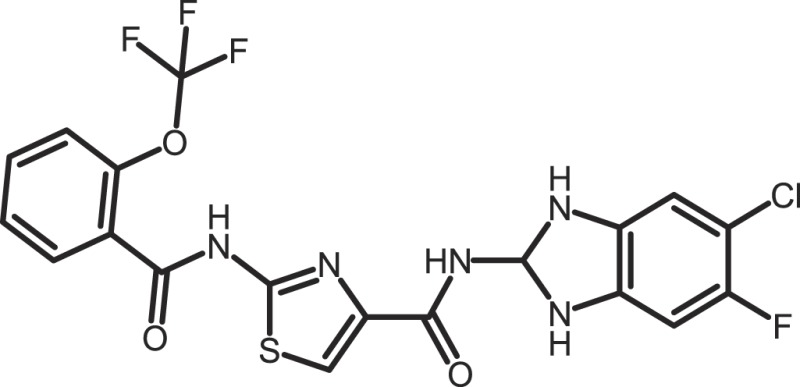	CK1δ: 0.042; CK1ε: 0.033	10	(367)
Hua-1h	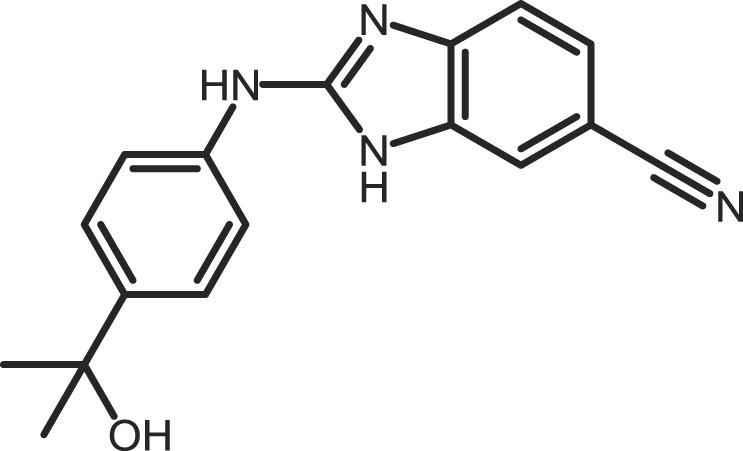	CK1γ: 0.018	Not reported	(368)
Yang-2	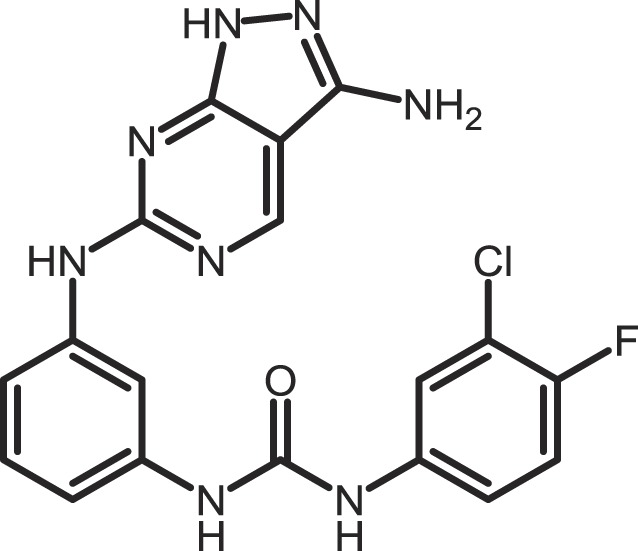	CK1: 0.078	Not reported	(369)
CK01	similar to PF-670462	Not reported	Not reported	(370)
MRT00033659	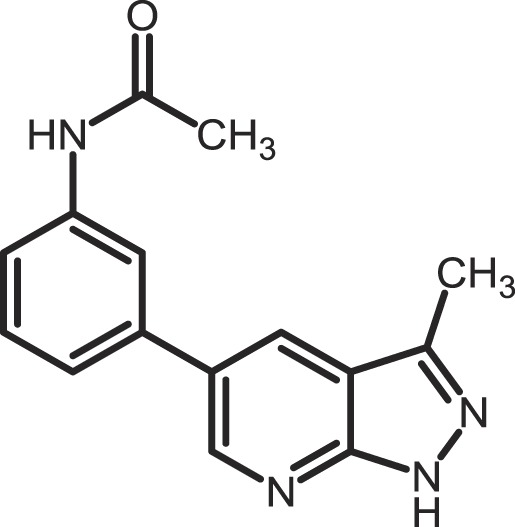	CK1δ: 0.8935	20	(371)
TG0003	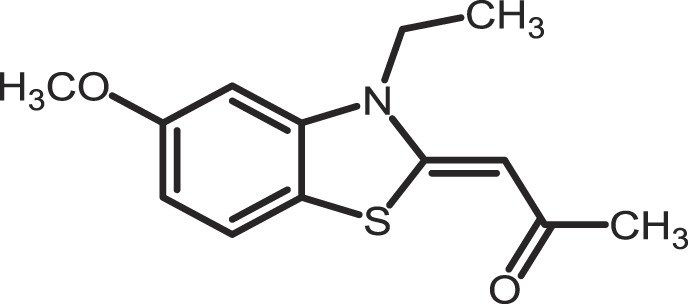	CK1δ: 0.4; CK1ε: 0.55	Not reported	(277, 372)
Salado-34	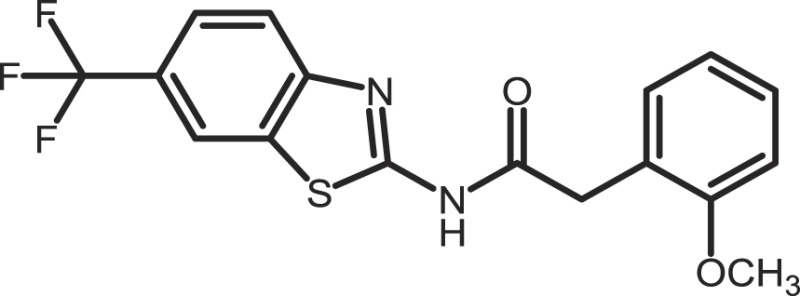	CK1δ: 0.01	10	(373)

**Table 4:** **Effects of CK1-specific inhibitors in selected animal models**.

Process	Inhibitor	Model	Effects	Reference
Circadian rhythm	PF-670462	Rat	Inhibition of CK1δ/ε yields perturbation of oscillator function leading to phase delays in circadian rhythms	(359)
	PF-670462	Rat	Chronic treatment with the CK1δ/ε specific inhibitor PF-670462 yields cumulative phase delays in circadian rhythms	(374)
	PF-670462	Monkey	Inhibition of CK1δ/ε produces phase shifts in circadian rhythms of Cynomolgus monkeys	(375)
	PF-670462; PF-4800567	Mouse	Whereas PF-670462 causes a significant phase delay in animal models of circadian rhythm, CK1ε-specific PF-4800567 only shows a minimal effect on the circadian clock	(273)
	CK01	Mouse	Chronic administration of CK01 leads to a reversal of the anxiety-related behavior, and partial reversal of the depression-related phenotypes of the Clock mutant mouse	(370)
	PF-670462; PF-4800567	Mouse	Selective inhibition of CK1δ acts as a potent *in vivo* regulator of the circadian clock and may represent a mechanism for entrainment of disrupted or desynchronized circadian rhythms	(278)
	PF-670462; PF-4800567	Zebrafish	The use of a pan-CK1δ/ε inhibitor and a CK1ε-selective inhibitor revealed that activity of CK1δ is crucial for the functioning of the circadian timing mechanism in zebrafish at multiple levels	(283)
Drug use disorder	PF-670462	Rat	Inhibition of CK1δ/ε in the nucleus accumbens with the selective inhibitor PF-670462 blocks amphetamine-induced locomotion by regulating of the AMPA receptor phosphorylation	(376)
Sensitivity to opioids	PF-4800567	Mouse	Co-administration of the CK1ε specific inhibitor of PF-4800567 increased the locomotor stimulant response to methamphetamine and fentanyl	(377)
Alcoholism	PF-670462	Rat	The inhibition of CK1δ/ε with systemic PF-670462 injections dose-dependently prevented the alcohol deprivation effect	(378)
Cancer	IC261	Mouse	Inhibition of CK1 isoforms by IC261 influences the growth of induced pancreatic tumors in SCID mice	(339)
	IC261	Mouse	IC261 treatment blocks MYCN amplified neuroblastoma tumor growth *in vivo*	(346)
	D4476	Mouse	Inhibition of CK1α activity leads to reduced Rps6 phosphorylation and activation of p53, resulting in selective elimination of leukemia cells	(348)
Spinal inflammatory pain transmission	IC261; TG003	Mouse	Both compounds decreased the frequency of spontaneous excitatory postsynaptic currents (sEPSCs) in inflammatory pain models	(379)

CKI-7 (*N*-(2-aminoethyl)-5-chloroisoquinoline-8-sulfonami de), was the first ATP-competitive inhibitor being described to show selectivity toward CK1 (356). Later, IC261 (3-[(2,4,6-trimethoxyphenyl)-methylidenyl]-indolin-2-one) and D4476 (4-[4-(2,3-dihydro-benzo)[1,4]dioxin-6-yl)-5-pyridin-2-yl-1*H*-imi dazol-2-yl]-benzamide) have been described as more potent and selective inhibitors, which also bind to the ATP binding pocket of CK1 (357, 358). Several effects reported for IC261-treated cells may however not be related to the selective inhibition of CK1 (380, 381). IC261 is also able to bind MT thereby inhibiting their polymerization similar to the spindle poison colchicine (380). Nevertheless, IC261 inhibits site-specific phosphorylation of p53 and Bid thereby inducing apoptosis in so-called type II cells (151, 187). Furthermore, its therapeutic potential has been demonstrated in xenotransplantation models for pancreatic cancer and neuroblastoma tumors (339, 346) (Table 4). However, it is still questionable whether the described anti-tumorigenic effects of IC261 are all mediated through selective inhibition of CK1δ and ε.

Two very potent and selective inhibitors for CK1δ and ε have been developed by Pfizer Global Research and Development: while PF-670462 possesses only poor isoform selectivity compound PF-4800567 shows a 22-fold stronger inhibition of CK1ε than CK1δ (273, 359). Furthermore, PF-4800567 demonstrated *in vivo* potency by altering the circadian clock in cycling Rat1 fibroblasts and in a mouse model for circadian rhythm (273). Recently, the use of PF-670462 (and the similar compound CK01) proofed to be beneficial in the treatments of bipolar disorder (370), addictive behavior (378), and in perturbed circadian behavior (278), respectively.

By using structure-based virtual screening Cozza and co-workers identified two amino-anthraquinone analogs as CK1δ-specific inhibitors (362). Furthermore, several roscovitine-derivatives, among them (R)-DRF053, have been shown to inhibit both CK1 and CDK family members (360). In 2009, imidazole- (compounds 17 and 18) and isoxazole-derivatives have been found to be highly potent inhibitors for CK1δ and ε (31). Furthermore, a 2-phenylamino-6-cyano-1*H*-benzimidazole derivate (compound 1h) was identified as CK1γ-specific inhibitor with excellent selectivity, cellular potency, and acceptable pharmacokinetic properties (368). A new lead compound (a *N*6-phenyl-1*H*-pyrazolo[3,4-d]pyrimidine-3,6-diamine derivative), which inhibits CK1 with an IC_50_ value of 0.078 μM was discovered by Yang and colleagues (369). By using a pyrazolo-pyridine analog as CK1/Chk1 dual-specific inhibitor the p53 pathway could be stabilized and reactivated (MRT00033659) (371). Benzimidazole-based CK1-specific inhibitors were reported by several recent studies (Table 3) [SR-3029 and SR-2890 (366), Bischof-5 and Bischof-6 (367), and Hua-1h (368)]. Furthermore, *N*-(benzothiazolyl)-2-phenyl-acetamides have been characterized as inhibitors for CK1δ-mediated phosphorylation of TDP-43 and may offer new therapeutic possibilities for the treatment of amyotrophic lateral sclerosis (ALS) (373). Quite recently, also the Clk-specific inhibitor TG003 was used to inhibit CK1 isoforms in a mouse model for mechanical allodynia and thermal hyperalgesia (Table 4) (379).

**Figure 9:**
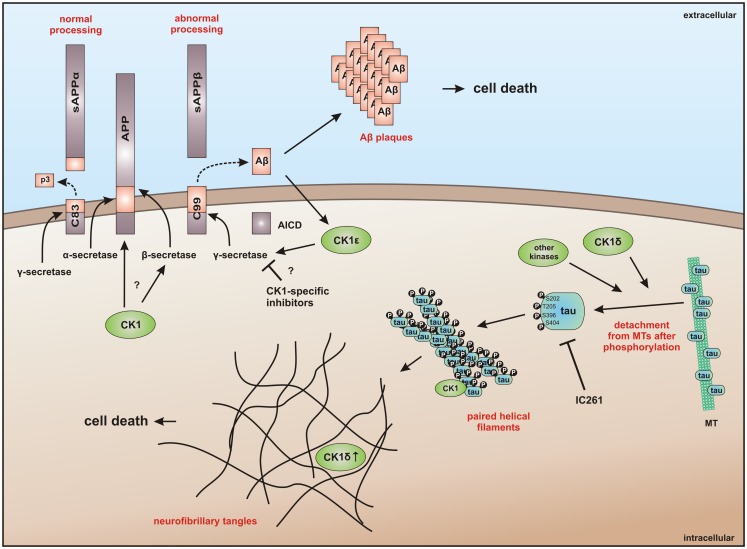
**CK1 in neuro-degenerative diseases**. It has generally been accepted that overexpression of CK1 plays an important role in neuro-degenerative diseases, especially in tauopathies, such as Alzheimer’s disease (AD). CK1δ is known to be up-regulated up to 30-fold on mRNA level in hippocampal regions of Alzheimer‘s disease (AD) brains (383). CK1δ plays a critical role in formation of neurofibrillary tangles through phosphorylation of tau at amino acids Ser-202/Thr-205 and Ser-396/Ser-404 (responsible for binding to tubulin) in human embryonic kidney 293 cells, thereby leading to a release of tau from MT and to destablilization of MT. Phosphorylation of these sites could be inhibited by the CK1-specific inhibitor IC261 (227). It is further known, that CK1 is associated to paired helical filaments in AD (384) and to tau-containing neurofibrillary tangles, in AD, Down syndrome, progressive supranuclear palsy, Parkinsonism–dementia complex and pallido-ponto-nigral degeneration (383, 385). The overexpression of constitutively active CK1ε, proposed to be involved in processing of amyloid precursor protein (APP) on γ-secretase level, results in an increase of amyloid-beta (Aβ) production, which is attenuated by use of CK1-specific inhibitors (386). In addition, Höttecke et al. (381) could show that the inhibition of γ-secretase by one of these inhibitors does not depend on CK1δ. An *in silico* analysis further revealed multiple CK1 consensus phosphorylation sites in the intracellular regions of APP, β-secretase, and γ-secretase subunits. Conversely, Aβ seems to influence CK1 activity (387). sAPPα/β: secreted amyloid precursor protein α/β; AICD: amyloid precursor protein intracellular domain.

The potential of CK1-specific inhibitors for the treatment of neuro-degenerative diseases, like AD and Parkinson’s disease, have been recently reviewed in detail by Perez and colleagues (382). The involvement of CK1 isoforms in the pathogenesis of AD is illustrated in Figure 9.

As an alternative to small molecule inhibitors lacking appropriate ADME (absorption, distribution, metabolism, and excretion) properties or showing unfavorable side effects synthetic peptides can also be used, which copy naturally occurring motifs that specifically influence the activity of the kinase or its interaction with cellular binding partners (388). Lately, small CK1α-derived peptides were used as *Biologic* tools to block CK1α binding to MDM2. At least, one peptide was identified to block the CK1α-MDM2 interaction (but not CK1α kinase activity) thus leading to decreased CK1α-MDM2-mediated degradation of p53 (208).

## Final Remarks

Summarizing the findings cumulated within many years regarding CK1 and its cellular functions, CK1 isoforms can be seen as central players in the regulation of numerous physiological cellular processes. Respecting this involvement in important cellular signal transduction pathways, it is reasonable that dysregulation of CK1 isoforms has been linked to the incidence of inflammatory and proliferative diseases but also to neuro-degenerative disorders. A summary of CK1-associated functions in neuro-degenerative diseases can be found in Figure 9 and its associated figure legend. If potent CK1 (isoform)-specific inhibitors were available new therapeutic possibilities for personalized medicine could be provided. However, the development of isoform-selective compounds available for *in vivo* application still remains challenging and inhibitor development should include not only conventional small molecule design, but also novel peptide inhibitor approaches.

## Authors Contribution

All Authors (Uwe Knippschild, Marc Krüger, Julia Richter, Pengfei Xu, Balbina García-Reyes, Christian Peifer, Jakob Halekotte, Vasiliy Bakulev, and Joachim Bischof) were involved in writing passages of the present review article and participated in final approval and revision. Figures and tables were created by Uwe Knippschild, Marc Krüger, Julia Richter, Pengfei Xu, Balbina García-Reyes, Christian Peifer, Jakob Halekotte, Vasiliy Bakulev, and Joachim Bischof.

## Conflict of Interest Statement

The authors declare that the research was conducted in the absence of any commercial or financial relationships that could be construed as a potential conflict of interest.
